# Perspectives of Microbial Inoculation for Sustainable Development and Environmental Management

**DOI:** 10.3389/fmicb.2018.02992

**Published:** 2018-12-05

**Authors:** Maqshoof Ahmad, Lisa Pataczek, Thomas H. Hilger, Zahir Ahmad Zahir, Azhar Hussain, Frank Rasche, Roland Schafleitner, Svein Ø. Solberg

**Affiliations:** ^1^Department of Soil Science, University College of Agriculture and Environmental Sciences, The Islamia University of Bahawalpur, Bahawalpur, Pakistan; ^2^Institute of Agricultural Sciences in the Tropics (Hans-Ruthenberg-Institute), University of Hohenheim, Stuttgart, Germany; ^3^Institute of Soil and Environmental Sciences, University of Agriculture Faisalabad, Faisalabad, Pakistan; ^4^World Vegetable Center, Tainan, China; ^5^Inland Norway University of Applied Sciences, Elverum, Norway

**Keywords:** biopesticides, phytoremediation, pollution, mitigation strategies, soil microbes, sustainability

## Abstract

How to sustainably feed a growing global population is a question still without an answer. Particularly farmers, to increase production, tend to apply more fertilizers and pesticides, a trend especially predominant in developing countries. Another challenge is that industrialization and other human activities produce pollutants, which accumulate in soils or aquatic environments, contaminating them. Not only is human well-being at risk, but also environmental health. Currently, recycling, land-filling, incineration and pyrolysis are being used to reduce the concentration of toxic pollutants from contaminated sites, but too have adverse effects on the environment, producing even more resistant and highly toxic intermediate compounds. Moreover, these methods are expensive, and are difficult to execute for soil, water, and air decontamination. Alternatively, green technologies are currently being developed to degrade toxic pollutants. This review provides an overview of current research on microbial inoculation as a way to either replace or reduce the use of agrochemicals and clean environments heavily affected by pollution. Microorganism-based inoculants that enhance nutrient uptake, promote crop growth, or protect plants from pests and diseases can replace agrochemicals in food production. Several examples of how biofertilizers and biopesticides enhance crop production are discussed. Plant roots can be colonized by a variety of favorable species and genera that promote plant growth. Microbial interventions can also be used to clean contaminated sites from accumulated pesticides, heavy metals, polyaromatic hydrocarbons, and other industrial effluents. The potential of and key processes used by microorganisms for sustainable development and environmental management are discussed in this review, followed by their future prospects.

## Introduction

Population growth and industrialization has put significant pressure on global ecosystems. Currently, 39% of terrestrial biomes are affected by intensive land use or settlements (Ellis et al., 2010). Urbanization often takes place on previously cultivated land and to produce more food, farmers tend to intensify, using agrochemicals that include a variety of structurally different compounds. This is especially predominant in developing countries (Lichtfouse et al., 2009).

Fifty years ago, the “green revolution” was launched, combining high-yielding cultivars, inorganic fertilizers, and pesticides to foster food production (Shelton et al., 2012). The impacts were great, albeit, today, to maintain healthy environments, new technologies need to be applied, including microbial inoculations (Garcia, 2012). These can either replace or reduce agrochemicals, and also clean areas heavily affected by pollution (Finley et al., 2010). Although green revolution and industrialization generally made human life easier and improved world's economy, effluents from industry also affected the health of soil, water and atmosphere leading to overall environmental degradation. Heavy metal contamination from industries poses deleterious effects not only on soil fertility and plant growth but also a serious serious threat to vulnerable human health (Oves et al., 2012). The over-use of ecosystems services is also alarming. While the use of regulating services, such as air quality, erosion control, and water purification as well as provisioning services, such as pollination and genetic resources strongly increase, their conditions steadily decrease (Carpenter et al., 2009). It is estimated that 22 million hectares of soil are adversely affected by chemical contamination worldwide, mostly in Europe, but also in Asia (Bai et al., 2008). The contamination and degradation of ecosystems by industrial pollutants is an emerging problem in the twenty-first century. There are a number of approaches, which can be used, on sustainable basis to meet food requirements without compromising environmental health. Among these, use of microbial products is pivotal to ensuring food security in changing climate (Timmusk et al., 2017). Microbial approaches can successfully be used for sustainable agricultural development. These microbes can enhance plant growth by improving nutrient availability to crop plants through various mechanisms (Zaidi et al., 2009) thus decrease the dependence on chemical approaches.

In this review, tradeoffs of the green revolution and potential uses of microbial inoculation technologies for sustainable development and environmental management are summarized. We discussed how microbes can be used as biofertilizers and biopesticides to reduce our dependence on agrochemicals. Then the role of microborganisms for bioremediation of contaminated sites, polyaromatic hydrocarbons, and other industrial effluents is discussed. Moreover, examples on successful use of microorganisms for healthier and cleaner environments without compromising crop productivity and industrialization have been included in the below sections. Finally, future prospects of using microbes for decontamination of pollutants are discussed.

## Boon and Bane of the Green Revolution

The demand for food by a rapidly increasing world population can only be fulfilled through increased crop production, but while also utilizing available resources in a sustainable way. One of the outcomes of the green revolution was the development and use of agrochemicals, which exponentially increased crop productivity (Jackson-Smith, 2010; Shelton et al., 2012). Today, agrochemicals still hold a strategic position (Lichtfouse et al., 2009), but a very high cost has been paid by soil systems and the environment. Agriculture has turned into a synthetic production system, dependent upon chemical inputs. This is also the case in many developing countries (FAO, 2013; Moharana et al., 2014). It is evident that sustainability and maintenance of soil productivity cannot be achieved by the use of agrochemicals alone. McKelvey et al. (2014) found high pesticide contamination in U.S. cities when non-experts applied pesticides to the environment non-discriminately. Often, only 0.1% of pesticides applied to farmlands actually reach their intended targets, the pests. The remaining 99.9% disperses or persists in the air, water, and soil, eventually entering our food chain (Aktar et al., 2009). Pesticide residues have also poisoned pristine environments far from where they have been used, such as the Arctic, the Antarctic, the Himalayas and the Great Barrier Reef as reviewed by Mullin et al. (2015). It is more challenging in developing countries, where large populations live in close proximity to farmland, often leading to direct exposure and severe health issues in humans (Watson, 2014).

Increasingly, pests have also developed resistance toward pesticides, and farmers are using higher doses to overcome declining yields. Extensive use of agrochemicals can not only lead to pesticide resistance, but also decrease profits for farmer, as more, at higher doses, are needed to control pests. Agrochemical use not only affects the soil and environment, but also beneficial organisms, such as birds and insects (Anderson et al., 2014). The disappearance of pollinators and birds is the result of uncontrolled use of agrochemicals and their persistence in the environment.

Even if agrochemicals are needed in the face of increasing concerns for food security, they have been shown to have potent impacts on both the environment and people, indicated by clusters of diseases, such as cancers, neurological diseases, fertility issues, and mental and physical disabilities (Iida and Takemoto, 2018). A reduction in agrochemicals use may lead to healthy crops and healthier environments (Frische et al., 2018).

## Microorganisms as Plant Growth Promoters

Plants are entirely dependent upon soil microorganisms to utilize soils as a growth medium, and the synergy between both is important for their survival (Rajendhran and Gunasekaran, 2008). The rhizosphere, the region of soil surrounding the roots, has the greatest concentration of microorganisms (Hiltner, 1904). Root exudates dictate the microbial communities. Manipulating the rhizosphere, changes microbial diversity and could improve plant performance by influencing water dynamics and enzyme activities (Ahmadi et al., 2018). A wide range of microscopic organisms inhabits the rhizosphere: bacteria, algae, fungi, protozoa and actinomycetes. Of these, bacteria is the most abundant and important group of microorganisms regarding plant growth and productivity (Ahmad et al., 2013a). They either live freely in rhizosphere, or in inter and intracellular spaces of root tissues, forming symbiotic associations with plants (Nadeem et al., 2014). Fungi play an important role in organic matter decomposition, and therefore nutrient cycling. Among soil fungi, arbuscular mycorrhizal fungi (AMF) are the most important and widely studied group as potential biofertilizers and biopesticides (Nadeem et al., 2014). Examples of use of soil microorganisms for improving crop growth and productivity have been summarized in Table 1.

**Table 1:** Effect of inoculation with soil microorganisms on plant growth.

**Crop**	**Microbial strain**	**Growth conditions**	**Effect on plant growth**	**References**
*Phaseolus vulgaris*	*Cellulosimicrobium funkei* KM032184	Greenhouse experiment/chromium stress	Enhanced seed germination rate, shoot and root length, total biomass, chlorophyll, and carotenoid contents by modulating oxidative damage	Karthik et al., 2016
Chickpea	*Pseudomonas aeruginosa* OSG41	Pot experiment/chromium stress	Increased dry matter accumulation, nodule formation, grain yield, and protein of chickpea	Oves et al., 2013
Rice	*Pseudomonas fluorescens* (PSF), *P. putida* (PSP)	Field experiment/normal soil	Significant improvement in growth, yield, and yield contributing parameters	Chamani et al., 2015
Maize (*Zea mays*)	*Klebsiella* sp., *Pantoea* sp., *Enterobacter* sp.	Pot trial/normal soil	Dual inoculation of endophytic and rhizobacteria improved growth and indole acetic acid contents in maize	Rodrigues and Forzani, 2016
Wheat	*Azospirillium* sp.	Field experiment/normal soil	Significant increase root length, and root fresh and dry weight	Singh et al., 2017
Carrot	*Pseudomonas syringae* pv. *syringae* Pss20 and *Pseudomonas tolaasii* Pt18	Laboratory experiment/abiotic stress	Inoculation showed biocontrol potential and significantly enhanced root formation of carrot slices	Etminani and Harighi, 2018
Cucumber (*Cucumis sativus*)	*Pseudomonas fluorescens*	Laboratory study/salinity stress	Improved root and shoot growth by reducing the negative effects of salinity stress	Nadeem et al., 2017
*Wedelia trilobata*	*Bacillus* sp. WtEB-JS040	Laboratory experiment/normal conditions	Significant improvement in growth of inoculated plants was observed	Dai et al., 2016
Century plant (*Agave americana* L.)	*Rhizobium daejeonense, Acinetobacter calcoaceticus* and *Pseudomonas mosselii*	Pot experiment/normal conditions	Inoculation significantly increased plant growth and sugar contents in century plant through nutrient solubilization and phytohormones production	Torre-Ruiz et al., 2016
Tomato	*Azotobacter chroococcum*	Salinity and drought stress	Improved plant growth by reducing the negative effects of stress on plants	Viscardi et al., 2016
*Camelina sativa*	*Pseudomonas migulae* 8R6	Pot experiment/salinity stress	Improved plant growth by reducing the effect of higher ethylene production through ACC-deaminase activity	Heydarian et al., 2018
Maize	*Azospirillum* sp. Az3, *Azospirillum* sp. Az8, *Azospirillum* sp. Az19, *Azospirillum* sp. Az63, *A. brasilense* Az39	Pot experiment/drought stress	Inoculation enhanced the drought tolerance in maize seedlings, and improved root and shoot growth	García et al., 2017
Wheat	*Arthrobacter protophormiae* (SA3), *Bacillus subtilis* (LDR2), and *Dietzia natronolimnaea* (STR1)	Hydroponic experiment/salinity and drought stress	Inoculation improved the salt and drought tolerance thus improved growth of wheat seedlings	Barnawal et al., 2017
*Citrus macrophylla*	*Pseudomonas putida* and *Novosphingobium* sp.	Pot experiment/salinity stress	Reduced the effects of salinity stress by decreasing the production of abscisic acid (ABA) and salicylic acid (SA) in plants	Vives-Peris et al., 2018
White clover	*Rhizoglomus intraradices, Diversispora versiformis* and *Paraglomus occultum*	Pot experiment/normal conditions	Inoculation significantly increased the nodulation, root growth and chlorophyll contents	Lu and Wu, 2017
Lettuce	*Funneliformis mosseae* and *Rhizophagus intraradices*	Pot experiment/normal conditions	Enhanced plant growth though improvement in Zn uptake	Konieczny and Kowalaska, 2016
*Citrus aurantifolia*	*Glomus etunicatum* and *Pseudomonas fluorescence*	Pot experiment/drought stress	Inoculation enhanced citrus growth by improving chlorophyll contents and photosynthetic activity of the plant	Shahsavar et al., 2016
*Morus alba*	*Acaulospora scrobiculata, Funneliformis mosseae*, and *Rhizophagus intraradices*	Pot experiment/normal conditions	Inoculation improved plant growth through improvement in chlorophyll contents, photosynthesis and stomatal conductance of plants	Shi et al., 2016
Hangbaiju *(Chrysanthemum morifolium)*	*Funneliformis mosseae* and *Diversispora versiformis*	Pot experiment/salinity stress	Inoculation improved salinity tolerance of plants, and enhanced root and shoot growth, and root N contents	Wang et al., 2017
Soybean	Arbuscular mycorrhizal (AM) fungi	Pot experiment/drought stress	Inoculation improved plant growth and mitigated the negative impact of drought stress	Salloum et al., 2017
Maize	*Funneliformis mosseae* and *Pseudomonas fluorescens*	Pot experiment/drought stress	Inoculation enhanced the vegetative and reproductive traits, N and P uptake, root colonization and grain yield of maize	Ghorchiani et al., 2018
Soybean	*Bradyrhizobium* sp.	Field experiment/normal conditions	Inoculation increased the N, P and S contents, and improved seed and straw yield of soybean	Raja and Takankhar, 2018
Soybean	*Bradyrhizobium* sp.	Field experiment/normal conditions	*Bradyrhizobium* sp. enhanced the growth and grain yield of soybean	Galindo et al., 2018
Soybean	*Bradyrhizobium* sp.	Field experiment/normal conditions	Inoculation with *Bradyrhizobium* sp. increased the phosphorus use efficiency, and N and P uptake of soybean plants	Fituma et al., 2018
Peanut	*Bradyrhizobium* sp.	Field experiment/normal conditions	Inoculation enhanced the plant N and P uptake, and nodulation in peanut	Argaw, 2018
Wheat	*Rhizobium* sp.	Pot experiment/normal conditions	Inoculation with *Rhizobium* sp. enhanced root and shoot growth of wheat	Kamran et al., 2017
Maize	*Azospirillum brasilense Rhizobium tropici*	Greenhouse study/normal conditions	Inoculation improved plant root and shoot growth, and nitrogen accumulation in shoot of maize plants	Picazevicz et al., 2017
Pea	*Rhizobium leguminosarum*	Pot experiment/normal conditions	Inoculation reduced the disease severity, and improved seed fresh and dry weight	Wienkoop et al., 2017
Wheat	*Azorhizobium caulinodans*	Axenic conditions	*Azorhizobium caulinodans* inoculation improved the number and weight of leaves and roots	Liu et al., 2017

Taking a closer look at the rhizosphere, plants continually secrete synthesized food through their roots, nourishing a diverse community of soil rhizobacteria that in turn can strongly influence plant development by performing vital functions for plant. They are allies of plants, governing several fundamental processes related to plant growth. One of the important functions of plant growth promoting rhizobacteria (PGPR) is phosphorus (P) solubilization in the soil (Zhang et al., 2011a). The dynamics of P in soils are complex. Its availability for plants is often totally dependent on phosphate solubilizing bacteria (PSB), heterotrophic bacteria that secrete organic acids, which solubilize fixed forms of P and release available forms into the soil solution (He et al., 2002). Other extensively studied plant growth promoting traits are nitrogen fixation, 1-aminocyclopropane-1-carboxylic acid (ACC), deaminase activity, nutrient solubilization, chitinase activity, and catalase activity (Khan et al., 2009a; Ahmad et al., 2011; Nadeem et al., 2014; Xiao et al., 2017).

Endophytic bacteria in plants were first reported by Darnel in 1904. They have since been reported to be associated with almost every plant species. Their role in a plant's life is integral and their presence is considered as vital for plant function as nutrients, sunlight, and water. It has been reported that about 10^5^ cfu of endophytic bacteria are present per gram of fresh root weight, and their diversity so large that 70–80% of them have yet to be identified, despite recent advances. Endophytes are a group of microorganisms living in stems, leaves and roots of plants, and perform important ecological functions during the plant's life. The most important are protection against pathogens, interaction with plant symbionts, eliciting plant defense mechanisms against environmental stresses, production of volatile substances, and nitrogen fixation. Endophytic bacteria are also known to produce allelopathic substances, which serve as biological control for different pests (González and Lopez, 2013). The combination of these growth-promoting effects enhances plant's immunity level against diseases and pests (Hayat et al., 2010; Nadeem et al., 2014).

Endophytic bacteria live within the plant cells either forming special structures, such as nodules in legumes, or those that cannot be visually identified (Beneduzi et al., 2013). Maximum colonization and population size has been observed in the roots. When isolated from other tissues, endophytic bacteria will first colonize roots before inhabiting other organs. Plant roots are colonized by a variety of bacterial species from different genera, such as *Bacillus, Paenibacillus, Burkhulderia, Azotobacter, Rhizobium*, and *Pseudomonas*, which simultaneously function together to synergistically promote plant growth (Maheshwari, 2013). It is not certain if less studied non-symbiotic endophytic bacteria have any potential role relating to plant growth promotion (Rosenblueth and Martinez-Romero, 2006).

Large reservoirs of essential nutrients are present in the environment, but are not directly available to plants. Soil bacteria play a vital role in making these nutrients available for plant utilization. For example, nitrogen (N), when deficient, seriously limits crop productivity, despite that 78% of the atmosphere is made up of this element. However, it can be fixed by endophytic bacteria, making it available to not only plants, but also the entire ecosystem. In soils, most N, as other elements are locked in organic matter. As a result, the role of bacteria as nutrient cyclers is essential (Rasche and Cadisch, 2013). Bacterial activity releases all organically bound nutrients into the ecosystem and continues the natural cycle and flow of raw material from source to sink. Similarly, other nutrients, such as P, potassium (K), sulfur (S), calcium (Ca), and magnesium (Mg) cannot be utilized by plants unless mineralized and made available for plant uptake. Exogenous application of microbes can help in enhanced colonization in roots, increasing the availability of nutrients, minimizing the use of chemical fertilizers, and conserving of organic systems (Perotti and Pidello, 2012; Ahemad and Kibret, 2014).

**Figure 1:**
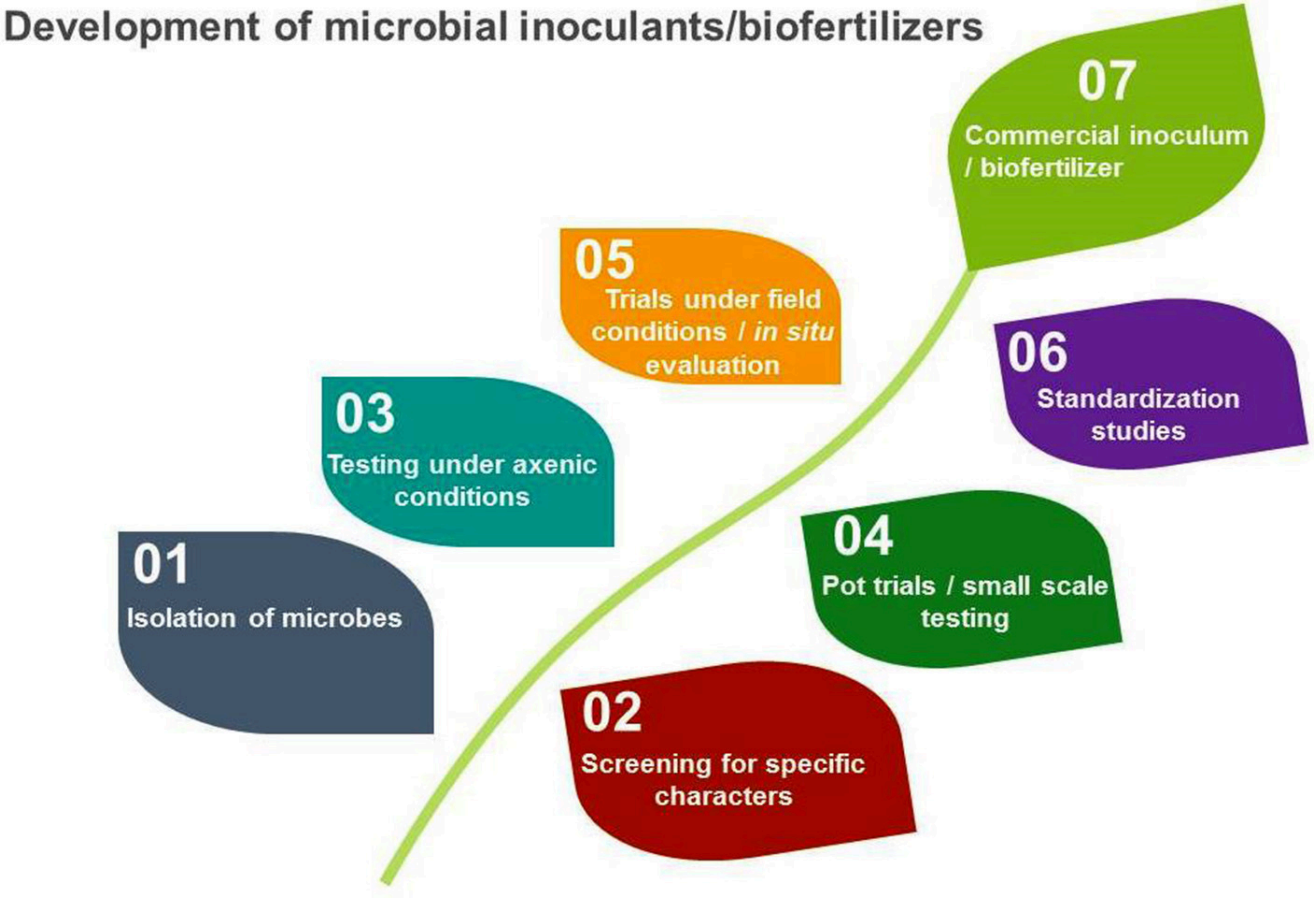
Stages in the development and commercialization of microbial inoculants/biofertilizers.

However, microbes used for these purposes are very specific and need to be screened for specific characteristics before development of biofertilizers. The development of microbial inoculants/biofertilizers is a highly technical and specialized job that goes through a number of steps before its ground level use (Figure 1).

The use of multi-strain bio-inoculants is more effective in establishing sustainable pest management solutions and solubilizing fixed nutrients. Rhizobacteria can be used in combination with endophytic bacteria and arbuscular mycorrhizal fungi, significantly enhancing crop production, and lessening dependency on fertilizers (Pérez et al., 2007). The combined use of endophytic bacteria and rhizobacteria is a novel approach, and recent reports showed that their application is highly effective. PGPR and plant endophytic bacteria are also highly useful in pest control, including insects, pathogens and weeds. Prabhukarthikeyan et al. (2014) found that combined use of PGPR and endophytic *Bacillus* bacteria effectively controlled fusarium wilt and fruit borer in tomatoes without pesticide application. Rhizobacteria are unique in their ability to control plant diseases. For example, Bandi and Sivasubramanian (2012) reported that *Pseudomonas fluorescens* has the ability to induce systemic resistance (ISR) against thrips (*Thrips tabaci* L.) and serve as biocontrol agent against pests.

Despite the use of microbes as biofertilizers, there is still a significant gap in the understanding of their role in agriculture and plant ecology. Their application has a significant positive impact on plant growth and productivity (Nadeem et al., 2011, 2014, 2015, 2016; Ahmad et al., 2016) through environmental acclimatization, enhanced resistance to pests, and improved stress resistance toward heavy metals, high salt concentrations, pathogens, and extreme pH.

Microbes, especially bacteria, produce allelopathic substances, which can serve as biological control against various pests (Sessitsch et al., 2004). The ability of microbes to synthesize metabolites that inhibit the activity of plant pathogens and prevent plant diseases indicates their potential as effective biopesticides. Studies have shown beneficial microbes in soil destroy pathogens, such as fungal, bacterial and viral diseases, insects, weeds, and nematode pests, through biocontrol or ISR (Gao et al., 2015). Many researchers focused on effects of microorganisms on seed germination and young weed seedlings (Harding and Raizada, 2015).

Specific endophytes play an important role in plant protection against soil borne pathogens (Sturz et al., 2000). Fungal pathogens are the most lethal to plants, but due to the antagonistic activity of hydrolytic enzyme producing bacteria their presence is rarely observed. Therefore, the application of bacteria as biopesticides could significantly reduce the use of agrochemicals for sustainable crop production. For example, in many crops like sugarcane, tomato, and potato, inoculation by rhizobacterial strains resulted in complete prevention of pathogenic development due to the production of antibiotic substances, resulting in ISR in crop species (Sessitsch et al., 2004). Bacteria are known to produce hydrolytic enzymes and binding proteins in plants that efficiently control bollworms, mosquitoes, blackfIies and beetles. Endophytic bacteria have been known to supress competing weeds through allelopathy effect, or by developing a synergistic relationship with bacteria and fungi in the rhizosphere (Bailey et al., 2010; Gao et al., 2015).

The use of microorganisms as biopesticides is an environmentally friendly approach, as these microbes are very specific to their host pathogens (Kachhawa, 2017). They could decrease agrochemical use, helping to foster environmental sustainability by reducing the harmful effects of toxic chemical compounds.

**Figure 2:**
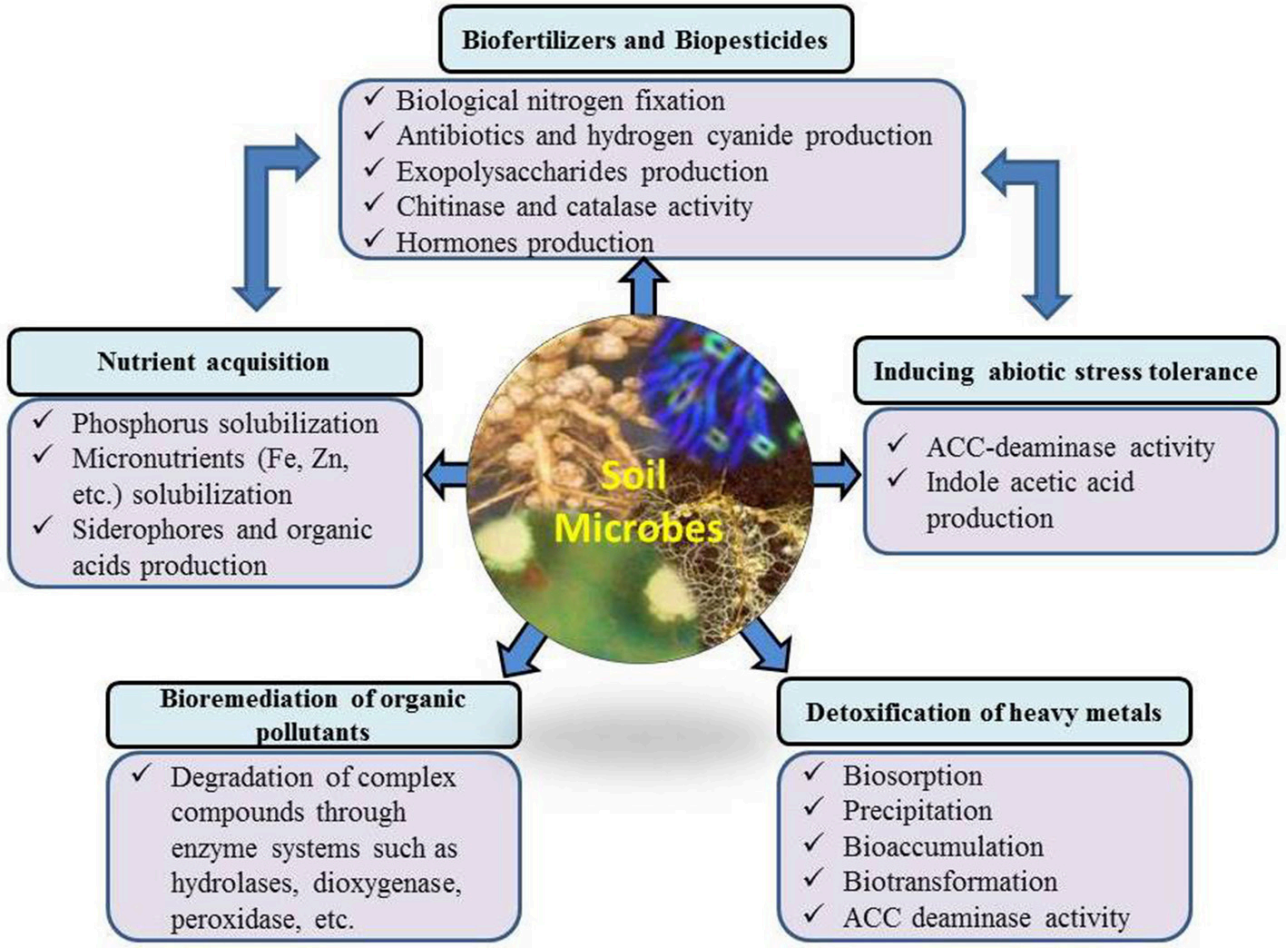
Importance of the microbial community for environmental health and possible mechanisms of action.

PGPR have direct and indirect plant growth promoting influences (Figure 2) through which they help plants to perform better under field conditions. Some of these effects are very common among culturable microbes, while others are specific to certain microbial strains/species. Under diverse environmental conditions, there are large fluctuations in microbial communities in the rhizosphere, influenced by plant species, soil moisture and temperature regimes, environmental conditions and soil physiochemical conditions (Galazka et al., 2017). For example, Gałazka and Grzadziel (2018) reported the fungal genetic diversity and community level through physiological profiling of microbial communities in the soil under long-term maize monoculture. They reported that techniques of maize cultivation and season had a great influence on the fungal genetic structure in the soil. These fluctuations in soil and environmental conditions also induce or suppress different plant growth promoting characteristics of microbial/strains. The most common direct effects include biological nitrogen fixation, phytohormone production, nutrient solubilization/mobilization, and siderophore production. Indirect effects include biological control of phytopathogens, and production of hydrolytic enzymes. PGPR are also effective in improving plant growth in stress conditions through ACC-deaminase activity, exopolysaccharides, production and scavenging toxic reactive oxygen species.

### Direct Effects on Plant Growth

The most important direct effects involved in plant growth promoting include biological nitrogen fixation, phytohormone production, nutrient solubilization, siderophore production, and ACC deaminase activity.

### Biological Nitrogen Fixation

Atmospheric N is reduced to plant available form though natural or artificial means. When done artificially, N_2_ is reduced to ammonia via the Haber–Bosch process (Rubio and Ludden, 2008), in which natural gas (CH_4_) and N_2_ are converted to reduced forms of N at high temperature and pressure. In nature, N_2_ reduction is performed by N-fixing microorganisms that use the nitrogenase enzyme to reduce N_2_ to ammonia (Kim and Rees, 1994). This biological nitrogen fixation (BNF) is responsible for two-thirds of the total fixed N worldwide.

The microbes performing BNF can be generally categorized as symbiotic, associative symbiotic, and free-living. However, a number of free-living N fixing bacteria, such as *Azotobacter, Gluconoacetobacter*, and *Azospirillum* spp. are present in nature and fix N for plants (Bashan and Levanony, 1990). The highest proportion of BNF is performed by symbiotic N_2_ fixers, i.e., rhizobia, which make symbiotic associations with the roots of leguminous plants (Zahran, 2001). The establishment of symbiotic association involves a complex mechanism and exchange of chemical signals between host plant and symbionts i.e., rhizobia (Giordano and Hirsch, 2004), leading to the formation of root knots, also called nodules. These develop from the swelling of cortical cells that host rhizobia as intracellular symbionts.

PGPR other than rhizobia also have the nitrogenase enzyme and can fix N in non-leguminous plants, such as diazotrophs, which are capable of forming non-obligate interactions with host plants (Glick et al., 1998) other than legumes. Nitrogenase is a two-component metallo-enzyme (Dean and Jacobson, 1992) that consists of an iron (Fe) protein (dinitrogenase reductase) and molybdenum (Mo)-Fe protein (dinitrogenase). For nitrogenase complex to function, both components should be present. During N fixation, the Fe protein receives electrons with high reducing power from a low redox donor, such as reduced ferredoxin (Fd) or flavodoxin, and is reduced itself. The reduced Fe protein passes its electrons to Mo-Fe protein and becomes oxidized. This complex chain of reactions requires significant metabolic energy to reduce N_2_. The genes involved are *nif* genes, which are present both in symbiotic and free-living microorganisms (Kim and Rees, 1994). Inoculation of legumes with rhizobia of a specific cross inoculation group can be helpful to improving the nodulating capability of crops under field conditions (Ahmad et al., 2013b).

### Phytohormone Production

Plants produce plant growth regulators/phytohormones, complex organic compounds that control plant growth and productivity. Due to their complexity, plants need a considerable amount of energy and nutrients to synthesize them. Bacteria synthesize significant quantities of phytohormones, and release them into the plant, resulting in pronounced positive effects on plant growth and development. It is reported that bacteria can produce up to 60 times more plant growth regulators than plants themselves (Camerini et al., 2008).

Phytohormones, such as indole acetic acid (IAA), ethylene, abscisic acid, cytokinins, and gibberellins production by PGPR help to improve crop growth and performance. Phytohormones are involved in plant growth at different scales, such as cell division, cell enlargement, seed germination, root formation, and stem elongation (Taiz and Zeiger, 2000; Khalid et al., 2006). Microbially-produced phytohormones have direct influence on plants' internal physiological processes and are involved in plant growth (Kang et al., 2010). The effectiveness of these microbially produced phytohormones to improve crop productivity has been well-documented (Zahir et al., 2007, 2010; Jamil et al., 2018). Microbes can meet the plant's hormonal requirements, saving the plant's metabolic energy for growth and reproduction. Microbially produced phytohormones play an effective role both under normal and stress conditions.

Auxins can alleviate the adverse effects of stress on plant growth. Some plants produce enough auxins to cope with adverse conditions, while others produce insufficient amounts, resulting in an inability to alleviate stress conditions. To meet the plant's auxin requirements, exogenous application of auxins or inoculation with microbes capable of producing auxins can be helpful and allow for resumption of normal metabolic functions. Ahmad et al. (2013c) evaluated the potential of auxin-producing *Pseudomonas* and *Rhizobium* strains to improve osmotic stress tolerance in *Vigna radiata* reporting an increase in total dry matter and salt tolerance index. In another study, Jamil et al. (2018) exogenously applied L-tryptophan in combination with *Pseudomonas fluorescens* under drought conditions, and reported a significant increase in growth, physiology, and yield. Abscisic acid (ABA) also improves plant development under stress conditions (Zhang et al., 2006) and plays an important role in photoperiodic induction of flowering (Wilmowicz et al., 2008). Patten and Glick (2002) inoculated canola plants with IAA-producing bacterial strains and reported increase in root length in comparison to IAA-deficient mutant and control plants. Similarly, the production of auxins, cytokinin and gibberellins by many strains of *Bacillus, Paenibacillus, Pseudomonas*, and *Azospirillum* has been reported (Ahmad et al., 2011; Gamalero and Glick, 2011; Mumtaz et al., 2017). Moreover, phytohormone-producing strains improved the growth and productivity of onion (*Allium cepa*) (Ahmad et al., 2016), cucumber (*Cucumis sativus*) (Ahamd et al., 2015) and maize (*Zea mays*) (Mumtaz et al., 2018). Steenhoudt and Vanderleyden (2000) showed that the production of phytohormones is the main mechanism used by *Azospirillum* strains to improve plant growth. Commercially available phytohormones can be used for improving plant growth (Jamil et al., 2018), although microbially-produced phytohormones are often more effective and economical under field conditions (Khalid et al., 2006).

### Nutrient Solubilisation

Soil microorganisms are solely responsible for nutrient cycling. Around 50% of soil organic matter is composed of carbon, while the rest consists of N, P, S, and other nutrients. In addition to the decomposition of soil organic matter, microbes also make chemically fixed nutrients, such as phosphorus (P), zinc (Zn), potassium (K), and iron (Fe) available. The main mechanism in the solubilization of P, K, Fe, and Zn is the lowering of pH from the production of organic acids (Jennings, 1994). The P solubilizing soil bacteria include free living rhizobacteria, such as *Pseudomonas*, the symbiotic nitrogen fixers (rhizobia), and asymbiotic nitrogen fixers (Azotobacter). In addition to release bound P through phosphatase production and rhizosphere acidification, these bacteria also provide phytohormones to crop plants and protect plants from various diseases through synthesizing siderophores, antibiotics, cyanogenic compounds, etc. (Khan et al., 2013). To solubilize Zn and phosphate, bacteria produce gluconic acid and its derivatives (Gadd and Sayer, 2000; Saravanan et al., 2007). Derivatives of gluconic acid e.g., 5-keto-gluconic acid and 2-keto-gluconic acid have also been reported in solubilization of Zn by Saravanan et al. (2007). Soil bacteria and fungi have ability to reinstate soil fertility of degraded lands by improving nutrient bioavailability through nitrogen fixation and solubilization of P, K, and Fe, and aggregate satbility (Rashid et al., 2016).

*Bacillus* spp. has the potential to solubilize insoluble Zn resources. Ramesh et al. (2014) demonstrated the Zn solubilization ability of *Bacillus aryabhattai* on Tris-minimal medium separately amended with different Zn compounds. Similarly, Hussain et al. (2015) reported the formation of a halo zone on ZnO amended Bunt and Revira medium by *Bacillus* sp. (AZ6). Mumtaz et al. (2017) also demonstated Zn solubilization by screening 70 isolates and identified four best ZSB strains, *Bacillus* sp. ZM20, *Bacillus aryabhattai* ZM31, *Bacillus subtilis* ZM63, and *Bacillus aryabhattai* S10. Gandhi and Muralidharan (2016) reported Zn solubilization in ZnO amended broth inoculated with *Acinetobacter* sp. (AGM3). Shaikh and Saraf (2017) reported a 6-fold increase in Zn and Fe content in grains of wheat inoculated with *Exiguobacterium aurantiacum* strain in comparison to the control. Solubilization of Zn in ZnO amended broth inoculated with *Bacillus aryabhattai* and *Gluconaacetobacter diazotrophicus* (Ramesh et al., 2014; Mumtaz et al., 2017) was also reported. *Bacillus* strains solubilize unavailable Zn by producing and secreting chelating ligands, organic acids, amino acids, vitamins, phytohormones, and through oxidoreductive systems and proton extrusion (Wakatsuki, 1995; Saravanan et al., 2003).

However, the processes involved in P solubilization depend on the organic and inorganic nature of phosphate complexes. Various biochemical processes, comprising of organic acids secretion and proton discharge, achieve phosphate solubilization. The extent and forms of fixed P in soil depend on the soil's pH. At lower pH, Fe, and aluminum (Al) phosphate complexes are abundant, while at higher pH, calcium phosphate minerals are formed (Goldstein, 2000). Solubilization of Fe/Al-phosphate complexes by PSB mainly takes place through proton release, altering the negative charge at exchange sites, to facilitate the release of phosphate ions. The decrease in adsorption of phosphates enhances availability of primary and secondary orthophosphates (Henri et al., 2008). Furthermore, PSB secrete carboxylic acid that releases carboxyl ions, which replace P in precipitated complexes through ligand exchange. In alkaline conditions, P is precipitated with calcium compounds (Goldstein, 2000). The PSB solubilize calcium phosphate complexes by releasing organic acids, acidifying the surrounding environment. The decrease in pH releases utilizable P and increases the availability of other macro and micronutrients. The calcareousness of soils increases buffering capacity, but reduces the efficiency of PSB in releasing P (Stephen and Jisha, 2009). To solubilize the organic proportion of immobilized P, PSB use alkaline and acidic phosphatases, which liberate bioavailable inorganic forms of P. Phosphatases are also exudated by plant roots, but the largest quantity is secreted by PSB (Dodor and Tabatabai, 2003).

Fe is an essential mineral nutrient in plants and microorganisms, except some species of lactobacilli (Neilands, 1995). Under aerobic conditions, Fe exists in its oxidized form, i.e., ferric Fe, which forms insoluble complexes, such as hydroxides and oxyhydroxides, which are unavailable for uptake by plants and microorganisms (Rajkumar et al., 2010). PGPR secrete low molecular mass iron chelating compounds, siderophores, which solubilize iron and increase its availability for microbes and plants (Machuca et al., 2007).

### Siderophore Production

Microbially-released siderophores increase plant Fe uptake through different mechanisms, such as chelation and release of Fe, direct uptake of Fe complexed siderophores, or by ligand exchange reactions (Schmidt, 1999). As a result, the soluble metal concentration increases through binding with siderophores. Inoculation with siderophore-producing rhizobacteria improved plant growth and nutrient assimilation (Rajkumar et al., 2010). Under low Fe conditions, siderophores can solubilize Fe from minerals and organic compounds (Khan et al., 2009a). Siderophores also form stable complexes with heavy metals, alleviating the toxic effect of heavy metals (Rajkumar et al., 2010). Marathe et al. (2015) inoculated *Glycine max* seeds with siderophore-producing *Pseudomonas* spp., and reported enhanced growth. Inoculation also showed antifungal activity against *Aspergilus* spp. In another study, Sharma et al. (2003) inoculated mung bean with *Pseudomonas* (GRP3) strain having ability to produce siderophores, and reported enhanced Fe contents in inoculated plants. Rajkumar et al. (2010) reported the uptake of Fe in cereal grains through production of siderophores by rhizobacteria. *Pseudomonas fluorescens* synthesizes Fe-pyoverdine, which can increase the Fe uptake in *Arabidopsis thaliana* tissues, enhancing plant growth (Vansuyt et al., 2007). Microbes with the ability to produce siderophores could be helpful in chelation of Fe from mineral and organic compounds to make it bioavailable. They also can promote uptake of Fe and other minerals in nutrient-deficient soils.

### ACC Deaminase Activity

Ethylene is an endogenously produced phytohormone with a specific role in determining plant maturity. Lower levels of ethylene are essential for plant metabolism during normal growth and development (Khalid et al., 2006). It is a stress hormone (Saleem et al., 2007) that helps plants to cope with biotic and abiotic stress. Ethylene negatively affects normal metabolic processes in plants leading to decrease in root and shoot growth. For example, Ahmad et al. (2011) reported a decrease in root and shoot length and increased stem diameter due to salinity stress and linked it to increased concentrations of ethylene. It has been well-documented that PGPR strains belonging to genera *Bacillus, Enterobacter*, and *Pseudomonas* isolated from stress conditions contain ACC deaminase enzyme (Nadeem et al., 2009; Ahmad et al., 2011) and improve plant growth under biotic and abiotic stresses (Mayak et al., 2004; Zahir et al., 2010; Ahmad et al., 2012, 2013b; Glick, 2012). ACC is the immediate precursor of ethylene and cleaves it into α-ketobutyrate and NH_3_ (Glick et al., 1998). Therefore, these bacterial strains can increase stress tolerance in plants by decreasing ethylene levels, allowing increased plant growth even under stress (Zahir et al., 2008; Ahmad et al., 2013b). Consequently, the use of these bacteria as biofertilizers and biopesticides can be helpful in reducing the dependence on chemical fertilizers and pesticides.

### Indirect Effects on Plant Growth

Examples of indirect effects of using soil microorganisms for plant growth promotion are biological control of phytopathogens through competition for nutrients, production of antibiotics, hydrolytic enzymes, and siderophores, along with the triggering of ISR in plants (Lugtenberg and Kamilova, 2009; Glick, 2012). PGPR can be used as a tool for biocontrol of plant pathogens as they indirectly improve plant growth by suppressing pathogenic microorganisms. The antibiotics produced are mostly effective against fungal pathogens (Glick, 2012). PGPR produce a number of antibiotics and antifungal metabolites, such as viscosinamide, tensin, pyrrolnitrin, phenazines, 2,4-diacetyphlaroglucinol, pyoluteorin and hydrogen cyanide (Raaijmakers et al., 2002; Haas and Keel, 2003; Compant et al., 2005; Mazurier et al., 2009; Bhattacharyya and Jha, 2012; Glick, 2012). Hydrogen cyanide works synergistically with antibiotics to improve their efficiency (Glick, 2012).

The production of hydrolytic enzymes, such as lipases, proteases, glucanases and chitinases by PGPR is also an effective mechanism of biocontrol. Hydrolytic enzymes dissolve the cell wall of fungal pathogens, suppressing their growth. Studies have shown PGPR strains to be successful in controlling pathogenic fungi from genera including *Fusarium, Sclerotium, Botrytis, Rhizoctonia, Phytophthora*, and *Pythium* (Frankowski et al., 2001; Kim et al., 2008; Glick, 2012).

In addition to improvement in Fe availability in crop plants, siderophores produced by PGPR strains can limit phytopathogen proliferation by suppressing Fe availability to pathogenic fungi (Kloepper et al., 1980). High affinity siderophores that bind Fe, limit its availability to fungal pathogens, therefore suppressing their growth (Glick, 2012). Siderophore producing bacteria can be used as a biocontrol agent, as plants need much lower Fe concentrations than most microorganisms (O'Sullivan and O'Gara, 1992) Moreover, many plants can take up siderophore-complexed Fe (Wang et al., 1993) while not the pathogenic microorganisms.

PGPR have the ability to effectively colonize plant roots to better use root exudates. Inoculation with PGPR can lead to higher proliferation and ultimately competition with pathogenic microorganism for nutrients and space, suppressing pathogenic microorganism growth. For example, Innerebner et al. (2011) tested bacterial strains from the genera *Methylobacterium* and S*phingomonas* for their biocontrol activity against *Pseudomonas syringae* pv. tomato DC3000 in *Arabidopsis thaliana*. They reported that strains from the genus *Sphingomonas* diminished disease symptoms, suppressed pathogenic growth, and protected *A. thaliana* plants from developing severe disease symptoms. However, *Methylobacterium* strains were ineffective in controlling pathogens.

PGPR interact with plant roots and induce resistance in plants against pathogenic microorganisms, leading to ISR. ISR is phenotypically similar to the systemic acquired resistance (SAR), the plant's internal mechanism for responding to infection by pathogens (Pieterse et al., 2009). In plants, ISR involves jasmonate and ethylene signaling, which stimulate the host plant's defense responses against a range of pathogens (Verhagen et al., 2004). In addition to phytohormones, individual bacterial components can induce ISR, such as homoserine, 2,4-diacetylphloroglucinol, cyclic lipopeptides, lipopolysaccharides (LPS), lactones, and some other volatile compounds (Lugtenberg and Kamilova, 2009).

## Microbial Remediation of Environmental Pollution

Anthropogenic activities and resulting waste disposal is a global issue, but is particularly problematic in regions with lax environmental regulations and legislation. In some developing countries, wastewater can in fact be highly desirable for farmers due to its high nutrient concentration. However, the long-term application of wastewater can alter the physical, chemical, and biological properties of soil (Narwal et al., 1988; Joshi and Yadav, 2005; Antil et al., 2007; Kharche et al., 2011) and lead to high concentrations of heavy metals (Narwal et al., 1988; Kharche et al., 2011) and dyes. Sewage sludge application on soils may, if not properly processed, contain potentially pathogenic organisms that pose health hazards (Chambers et al., 2001; Lapen et al., 2008; Edwards et al., 2009; Gottschall et al., 2009). Sludge, however, can be a rich source of nutrients, particularly for highly depleted soils. It contains N and P along with high concentration of organic matter, but may also contain heavy metals and chlorinated hydrocarbons (Jang et al., 2010).

Another potential industrial waste product is crude oil. Crude oil and other petroleum-based product spills can occur during transport and storage of petroleum products on land, but can also be spilled from tankers, offshore platforms, pipelines, drilling rigs, polluting soil, and water (Adelana et al., 2011). Petroleum products are a mixture of various organic compounds, many carcinogenic. Benzene, a petroleum product, is known to cause leukemia in humans (Kirkeleit et al., 2006).

The increase in heavy metal contamination worldwide is attributed to anthropogenic activities. Around the world, toxic effluents from industry and urban centers are polluting soil, air, and water, making them unfit for crop production, as well as human and animal well-being (Wahid et al., 2000). There is an urgent need to treat these industrial effluents to remove contaminants prior to discharge into the surrounding soil and water bodies. Attention has been given to remediation strategies of these pollutants due to their persistent nature and increased awareness among the global community (Ali, 2010). Several physicochemical methods have been used to detoxify industrial effluents (Arslan-Alaton, 2007). However, these methods are expensive and not environment friendly as they generate large amounts of sludge, which also requires safe disposal and can also cause secondary pollution (Zhang et al., 2004).

Microorganisms are being used for removing the pollutants from environment. These microbes can make a significant impact by removing contaminants from soils and reducing their toxic effect on the environment (EPA, 2016). The living microorganisms or their metabolites are used naturally or artificially to destroy, remove, or immobilize the pollutants from the environment (Uqab et al., 2016). PGPR can be successfully used in reenforcing plant growth by remediating contaminated and degraded soils and water bodies and removing toxins from the environment (Gouda et al., 2018).

Microorganisms contain enzyme systems with the potential to mineralize industrial effluents under different environmental conditions (Pandey et al., 2007). Bioremediation is a strategy that relies on the metabolic capabilities of microbes to transform aromatic compounds into essentially harmless or at least less toxic compounds (Xu and Zhou, 2017). It has a number of advantages compared to physicochemical methods. For example, microbial treatment is effective to degrade persistent and recalcitrant compounds. Other advantages include a reduction in sludge production, shorter treatment time, applicability over a wide range of temperatures, and are easy and simple to handle (Kulshrestha and Husain, 2007). It is based on the use of microorganisms with the ability to degrade recalcitrant and xenobiotic materials present in industrial wastewater.

Spina et al. (2018) emphasized the importance of fungi for biodegradation of pesticides and pollutants. They argue that currently perceived limitations of bioremediation, such as nature of organisms, the enzymes involved, the concentration, availability, and final survival of microorganisms, as well as the cost vs. overall environmental impact, can be solved to some extent by understanding the genetics and biochemistry of the desired microbes. The advent of synthetic communities indicated a sustainable way to facilitate the bioremediation as degradative fungi appeared to be particulary effective.

### Organic Pollutants

Persistant organic pollutants (POPs) include dyes used in textile and other inductries, pesticides and polycyclic aromatic hydrocarbons. These are toxic chemicals that have adverse effects on human health and environment around the world. Most of the POPs are generated in one country and affect human and wildlife far from where they are generated, used or released. Chemically these are complex compounds which are classified as xenobiotics thus barely removed from the environment. Microbial strains have been screened and identified that can degrade these POPs through their enzyme systems.

### Dyes

Bacterial strains have been identified that efficiently degrade the xenobiotic/recalcitrant compounds in industrial wastewater (Khalid et al., 2008a,b). Fungal species are also reported to degrade/detoxify industrial effluents. Bacteria contain specific enzymatic/gene systems responsible for the degradation of toxic compounds found in industrial effluents. Bacteria use biosorption and enzymatic degradation, or a combination of both, to detoxify/decolorize industrial effluents containing azodyes (Wu et al., 2012). An azo-reductase enzyme has been isolated and characterized from bacterial species. Azo-dyes have strong bonding properties, but some bacteria have the ability to break them with the azo-reductase enzyme (Chen, 2006). Oxygenase and hydroxylase enzymes also degrade the intermediate products formed during decolorization (Khalid et al., 2009). In this process, different amounts and kinds of microbes that degrade recalcitrant/xenobiotic compounds are involved (Table 2).

**Table 2:** Potential of microbial strains for detoxification of industrial effluents.

**Microbial strain**	**Experimental conditions**	**Response/Results**	**References**
*Brevibacillus laterosporus* MTCC 2298	Mixture containing seven commercial textile dyes with different structures and color properties	It showed 87% decolorization in terms of ADMI (American Dye Manufacturing Institute) removal within 24 h	Kurade et al., 2011
*Aspergillus niger*	Used pulp and paper industry wastewater	Decolorization of pulp and paper industry wastewater	Kamali and Khodaparast, 2015
*Aspergillus foetidus*	Used different textile effluents	Maximum decolorization of several azo dyes	Sumathi and Phatak, 1999
*Pseudomona putida*	Degradation of textile effluents observed at 25°C	Industrial textile wastewater, 95% color, 92% COD	Babu et al., 2011
*Pseudomonas* sp. SUK1	Sulfonated azo dye (Reactive Red 2) in a wide range (up to 5 g L^−1^), at temperature 30°C, and pH range 6.2–7.5 in static condition	Showed decolorization of the media containing a mixture of dyes	Kalyani et al., 2008
*Bacillus* *licheniformis* LS04	Reactive black 5, reactive blue 19 and indigo carmine was used in the experiment	More than 80% of color removal in 1 h at pH 6.6 or 9.0	Lu et al., 2012
*Tinctoporia borbonica*	Pulp and paper industry wastewater	Decolorization of pulp and paper industry waste	Senthilkumar et al., 2014
*Bacillus* sp. ADR	Used different azodyes	Decolorized different azo dyes with efficiencies of 68–90%	Telke et al., 2011
*Penicillium oxalicum* SAR-3	18S and internal transcribed spacer (ITS) rDNA gene sequence analysis and checked the degradation rate	Dye decolorization was detected	Saroj et al., 2014
*Bacillus* and *Pseudomonas*	Metals contaminated sites	Reducing the toxicity and concentrations of pollutants	Fosso-Kankeu et al., 2011
*Lenzites elegans* WDP2	Used different azodyes	Decolorized brilliant green, malachite green and congo red by 93, 21, and 99%, respectively	Pandey et al., 2018

Several studies demonstrated the ability of fungi, algae, and yeast to degrade industrial effluents in wastewater (Dresback et al., 2001; Olguin, 2003). However, carbon sources are required in the dye solution for microbial growth during the decolorization process. Factors, such as pH, temperature and presence of salts in contaminants can also affect the rate of the biodegradation of industrial effluents (Prasad and Rao, 2011). Many species of bacteria, fungi, algae, and yeast are reported to decolorize the dyes (Khalid et al., 2009).

Pandey et al. (2018) tested the potential of the lignolytic mushroom *Lenzites elegans* WDP2 to dicolorize synthetic dyes. Brilliant green, malachite green, and Congo red were decolorized by almost 93, 21, and 99%, respectively. The Congo red dye was completely bio-absorbed by fungal culture within 1 month. The fungal decolorized broth revealed an extracellular laccase activity in all the three cases, supporting the involvement of laccase enzyme in decolorization. Photomicrographs clearly showed the bio-sorption of the dyes by fungal culture into the mycelium/spores.

Paper and pulp industries also produce wastewater with toxic compounds. This is problematic because of the color of the water, toxic complexes, suspended solids, lignin, chlorinated compounds, chemical oxygen demand, and biological oxygen demand. Xenobiotics of paper, pulp, and mill effluents can be degraded by different microorganism including bacteria, fungi and actinomycetes (Hossain and Ismail, 2015).

*Bacillus cereus* and two strains of *Pseudomonas aeruginosa* have been found to decolorize bleached kraft paper-mill effluents (Tiku et al., 2010). *Pseudomonas putida* and *Acienetobacter calcoaceticus* degrade black liquor from a kraft pulp and paper mill. Their color removal efficiency was around 80% after 8 days (Abd El-Rahim and Zaki, 2005). A few algal species, such as *Microcystis* spp., can decolorize diluted bleached kraft paper-mill effluents. *Microcystis* spp. removed 70% color within 2 months (Sharma et al., 2014). Research is required to determine the pathways to degrade industrial effluents with microbial strains. Moreover, research focusing on the use of mixed cultures for the detoxification of these compounds should also be prioritized. The *in situ* application of this novel technology should also be further evaluated.

### Polycyclic Aromatic Hydrocarbons

Polycyclic aromatic hydrocarbons (PAHs) are organic pollutants, mostly originating from anthropogenic activities. Most are carcinogens and mutagens. Their longevity in terms of their persistence in the environment may be due to their complex structure, hindering their bioavailability. PAHs also have stronger adsorption potential on solid particles due to high hydrophobicity and solid-water distribution ratios (Johnsen et al., 2005). As a result of these qualities, they are difficult to degrade and pose a severe and long-lasting hazard to the environment. Long term contamination of petroleum products affects the microbial population and diversity in the soil (Galazka et al., 2018). One effective strategy is bioremediation, or the use of microbes for decontamination of complex organic compounds. Studies on the use of microbes for degradation of PAHs are summarized in Table 3.

**Table 3:** Microbial strains tested for degradation of polyaromatic hydrocarbons.

**Microbial strain**	**Experimental conditions**	**Response/Results**	**References**
*Pseudomonas plecoglossicida strain* PB1 and *Pseudomonas* sp. PB2	Naphthalene, fluoranthene, pyrene and chrysene in the balch tubes in liquid culture media	*Pseudomonas plecoglossicida* strain PB1 and *Pseudomonas* sp. PB2 degraded pyrene between 8 and 13%, chrysene 14 and 16%, naphthalene 26 and 40%, fluroanthene 5 and 7%, respectively	Nwinyi et al., 2016
*Bordetella avium* MAM-P22	Screened four naphthalene concentrations to determine the most potential strain having ability to use naphthalene as sole source of carbon and energy	*Bordetella avium* MAM-P22 degraded naphthalene to give six intermediate compounds viz. 1,2-Benzene dicarboxylic acid, Butyl-2,4-dimethyl-2-nitro-4-pentenoate, 1-Nonen-3-ol, Eicosane, Nonacosane	Abo-State et al., 2018
*Thalassospira* sp. strain TSL5-1	Pyrene degradation in the presence of additional nutrients, different pH and salinity levels	Optimum salinity level for degradation was 3.5% and 5%, pH fluctuation affected degradation rate and peptone had antagonistic effect with pyrene degradation	Zhou et al., 2016
Bacteria; *Pseudomonas* sp. N3 and *Pseudomonas monteilii* P26 and Actinobacteria; *Rhodococcus* sp. F27, *Gordonia* sp. H19 and *Rhodococcus* sp. P18	Pyrene, naphthalene and phenanthrene degradation at flask scale	Pure culture of *Pseudomonas* sp. N3 and *Pseudomonas monteilii* P26 efficiently degraded low molecular weight (LMW) PAHs but showed unfavorable results for degradation of high molecular weight (HMW) PAHs Actinobacteria; *Rhodococcus* sp. F27, *Gordonia* sp. H19 and *Rhodococcus* sp. P18 degraded relatively efficiently HMW PAHs but not able to degrade LMW PAHs The combination of four strains degraded phenanthrene and naphthalene (100%) while pyrene (42%); almost 6-times higher than pure cultures	Isaac et al., 2015
*Acinetobacter* sp. WSD	Biochemical characterization and 16S rDNA gene sequence analysis in the laboratory	The bacteria degraded phenanthrene (90%), fluorine (90%) and pyrene (50%), and used as sole source of carbon and degraded other PAHs as well after 6 days of incubation Glucose and humic acids improved degradation rate and served as alternate carbon source but co-metabolism was recorded in case of humic acid	Shao et al., 2015
*Amycolatopsis* sp. Poz14	Biochemical characterization and 16S rDNA gene sequence analysis in the laboratory, incubation studies	Utilized LMW; anthracene and naphthalene, and HMW; fluoranthene and pyrene polyaromatic hydrocarbons as sole source of energy and carbon Degraded naphthalene (100%), anthracene (37.9%), pyrene (25.1%), and fluoranthene (18.%) within 45-days of incubation	Ortega-Gonzalez et al., 2015
*Pseudomonas* sp. CES	Multiplexed LC-MS/MS assays in the laboratory, stable isotope dimethyl labeling	Bacterium able to degrade caffeine and can tolerate caffeine three times higher (9.0 g L^−1^) than the maximum tolerable levels of previously reported bacteria Discovered caffeine-degrading enzymes in bacterial strain	Yu et al., 2015
Three fungal strains: A; *Trichoderma/Hypocrea*, B and C; *Fusarium*	18S ribosomal DNA sequencing and morphological characterization incubation studies of 7 and 14 days at 28°C with PAHs as the substrate	Fungal strains used pyrene as the sole source of carbon strains A and B assimilated anthracene and fluoranthene, while strain C was unable to assimilate them Additional carbon source and pH affected the degradation potential and it was more at pH 4.0 than pH 6.5	Mineki et al., 2015
*Pleurotus ostreatus, Collybia* sp., *Rhizoctonia solani* and *Trametes versicolor*	Studied the potential ligninolytic activity using decolorization of a polymeric dye Poly R-478, ligninolytic enzyme profile studies	All fungi produced MnP and laccase while the *Collybia* sp. and *R. solani* also produced LiP in addition *T. versicolor* produced MnP and laccase 3–4 times more than the other fungi	McErlean et al., 2006
Consortium of five microorganisms. viz*. Achromobacter insolitus, Bacillus licheniformis, Bacillus cereus, Microbacterium* sp. and *Sphingobacterium* sp.	Studied the bioremediation of selected PAHs (naphthalene, anthracene and phenanthrene) by by shake flask method using microbial consortium isolated from petrochemical contaminated soil	Microbial consortium effectively biodegraded the naphthalene and anthracene. Microbial consortia consisting of these potential microorganisms can be used for biodegradation PAHs compounds generated by petrochemical industries	Fulekar, 2017
*Bjerkandera adusta, Pleurotus ostreatus* and *Phanerochaete chrysosporium*	Reviewed the biodegradation of PAHs by fungal enzymes	Fungal strains have the ability to produce ligninolytic enzymes, such as laccase, Mn peroxidase and lignin peroxidase applicable to PAH degradation	Kadri et al., 2016
*Pseudomonas gessardii strain* LZ-E	Studied simultaneous degradation of naphthalene and reduces Cr(VI) in aerated bioreactor system	*Pseudomonas gessardii* strain LZ-E continuously remediated naphthalene and reduced Cr(VI) to Cr(III)	Huang et al., 2016

However, PAHs can serve as energy source for microorganisms and consequently be converted into harmless or less toxic compounds. Bioremediation of PAHs is well-documented as cost-effective, feasible, and practically applicable natural process for the degradation of polyaromatic hydrocarbons in soil and water environments (Anwar et al., 2016). A variety of microorganisms, such as bacteria, fungi and actinomycetes can be used for bioremediation. However, bacteria are the most reliable in degrading PAHs in aquatic environments (Johnsen et al., 2005).

In sediments or soils, the efficiency of bacteria may be lower because of reduced bioavailability (Yuan and Chang, 2001), or alternatively improved due to the involvement of solid-phase bacteria in the degradation of sorbed contaminants (Hwang et al., 2003). The latter argument is supported by the work of Poeton et al. (1999), which showed the enhanced biodegradation rates of PAHs (phenanthrene and fluoranthene) by marine bacteria in the presence of sediments. However, further studies need to be conducted in order to evaluate the efficiency of bacteria in bioremediation of PAHs under a range of conditions. The effect of high sediment contents on biodegradation of PAHs in natural water bodies was studied by Xia et al. (2006). An increase in the population of PAH degrading bacteria in a water system corresponded with increasing sediment content.

PAHs meant to be degraded by bacteria must also be bioavailable (Dandie et al., 2004; Fredslund et al., 2008). Dissolution and vaporization make them bioavailable, but sorption on soil particles makes it difficult for bacterial degradation due to the limitations of the microbes under these conditions (Kim et al., 2007). The bioavailability of PAHs is affected by a number of factors, including soil structure, moisture contents, and presence of bacterial species. Uyttebroek et al. (2007) reported that desorption and ultimately decomposition of PAHs is affected by their age in soil. The rate of decomposition is also affected by the presence of readily available nutrient and carbon sources. Decomposition of PAHs becomes more difficult if the enzymes involved are non-specific. The enzymes would prefer to attach to substrates, which are easier to degrade, allowing PAHs to persist in the environment (Wang et al., 2009). The presence of intermediate compounds can favor decomposition of PAHs. *Sphingomonas, Nocardia, Beijerinckia, Paracoccus*, and *Rhodococcus* were found by Teng et al. (2010) to decompose anthracene completely in the presence of dihydriol, an initial oxygenated intermediate compound.

PAHs can also be degraded by fungal enzymes. Various aerobic and anaerobic fungi can be used for the degradation of PAHs (Aydin et al., 2016). *Bjerkandera adusta, Pleurotus ostreatus*, and *Phanerochaete chrysosporium* have the ability to produce ligninolytic enzymes applicable to PAH degradation, such as laccase, Mn peroxidase and lignin peroxidase (Kadri et al., 2016). The rate of degradation depends on culture conditions, such as the presence of oxygen, temperature, availability of nutrients, and in an agitated or shallow culture. The efficiency of fungal enzymes is also affected by addition of biosurfactants. The *in situ* biodegradation of PAH is more complicated due to heterogenic nature of soils (Kadri et al., 2016). Therefore, all factors affecting the bioavailability of PAHs should be considered when using fungi for the degradation of these compounds.

The PGPR also have the potential to degrade PAHs. *Pseudomonas* sp. JPN2 is able to degrade pyrene and other aromatic contaminants (Jin et al., 2016). Its degradation potential increased with increasing incubation time, and maximum degradation (83%) was achieved after 25 days of incubation. The analysis of culture medium on GC-MS showed four metabolites (1-hydroxy-2-naphthoic acid, 4-phenanthrol, 4,5-dihydroxy-4,5-dihydropyrene and phthalate). Additionally, JPN2 showed increased growth at 100 mg L^−1^ of pyrene, indicating its tolerance for pyrene (Jin et al., 2016). Meena et al. (2016) isolated pyrene degrading bacteria from industrial effluent contaminated sites which suggest these sites may be attractive niches for PAH degrading bacteria. From the industrial effluent, six bacterial isolates were isolated and purified following serial enrichment techniques, and were assessed for their pyrene degradation potential using modified mineral salt medium supplemented with pyrene. Through 16S rRNA sequencing, the isolated bacteria were found to belong to four genera, i.e., *Ochrobactrum, Microbacterium, Bacillus*, and *Acinetobacter*. The isolate *Bacillus megaterium* YB3 was further evaluated for its efficiency to degrade pyrene. Over 7 days of incubation, it degraded almost 73% of 500 mg L^−1^ pyrene into two relatively less toxic intermediate metabolites, based on GC-MS analysis. A further characterization of *B. megaterium* YB3 showed that it was positive for aromatic-ring-hydroxylating dioxygenase indole-indigo and catechol 1,2-dioxygenase conversion assays.

Bacteria belonging to the genus *Mycobacterium* are among the most effective degraders of PAHs. Hennessee and Li (2016) studied four *Mycobacterium* species with respect to single and mixed culture PAH metabolism. Four PAH compounds (phenanthrene, fluoranthene, pyrene, and benzo[a]pyrene) were used as model compounds to characterize the degradation potential of bacteria in a strain- and mixture-dependent manner. The results indicated metabolic differences between single and mixed degradation of PAH, which could be helpful for risk assessment and bioremediation of PAH-contaminated sites. Chen et al. (2016) investigated functional microbial populations and processes involved in pyrene biodegradation in soils. Two soils were incubated with 60 mg kg^−1^ of pyrene, more than 80% of the added pyrene degraded within 35 days in both soils. Thirty-five days after incubation, a significant enrichment of gram-positive bacteria harboring PAH-ring hydroxylation dioxygenase (PAH-RHD_α_ GP) genes was observed along with an increase in *Mycobacterium*. Both soils showed a large proportion of uncultured gram-positive bacteria along with *Mycobacterium*, suggesting they may be the important groups of pyrene degraders in soils. The genes, *nidA* and *nidA3*, were identified as the major genes involved in biodegradation following two different pyrene catabolic pathways (Chen et al., 2016).

The above studies point to the viability of microbes to degrade PAHs. Both bacteria and fungi are able to degrade various organic contaminants, including pesticides, azodyes, and petroleum hydrocarbons. In some cases, microbes use PAHs as energy and nutrient sources, converting them into harmless or less toxic compounds. In some cases, they rely on alternate energy sources and degrade pollutants without any direct benefits. Therefore, the addition of an alternate carbon source may accelerate the bioremediation process. Bioremediation is equally effective in liquid (water) and solid (soil) environments. However, it is affected by different biotic and abiotic factors, such as the type of microorganism involved, microbial population and diversity, structure of substrate, bioavailability of substrate, pH, aeration and alternate energy sources (Sihag et al., 2014). Microbes have different genes related to the degradation of PAHs, and consequently follow different pathways for decomposition of these compounds (Table 4). A number of intermediate compounds are produced depending upon the nature of the original pollutant and the type of microbe involved. These intermediate compounds can be even more toxic, therefore the bioremediation system should be continuously monitored. Although a lot of work has already been carried out in the field, more extensive studies using mixtures of contaminants and microbial consortia are needed. Further exploration of the specific mechanisms and genes involved needs more attention.

**Table 4:** Pathways used by microbial strains for the degradation of polyaromatic compounds.

**Microbial strain**	**Isolation source**	**Mechanism/pathway for degradation**	**References**
*Thalassospira* sp. strain TSL5-1	Coastal soil of Yellow Sea, China	Two pathways: salicylic acid and phthalate routes	Zhou et al., 2016
*Acinetobacter* sp. WSD	PAHs contaminated groundwater from a coal-mining area	Phthalic acid and Phenol, 2,5-bis(1,1-dimethylethyl) pathways	Shao et al., 2015
*Pseudomonas* sp. CES	PAHs contaminated area	Caffeine-degrading pathway	Yu et al., 2015
*Amycolatopsis* sp. Poz14	Oil-contaminated soil	Salicylic acid and phthalic acid pathways	Ortega-Gonzalez et al., 2015
*P. chrysosporium*	Pre-isolated obtained from, Institute of Microbiology, Chinese Academy of Science	Manganese peroxidase (MnP) and lignin peroxidase (LiP) pathways	Wang et al., 2009
*Pleurotus ostreatus, Collybia* sp., *Rhizoctonia solani* and *Trametes versicolor*	Grassland soil	Ligninolytic enzymes production, MnP and laccase production	McErlean et al., 2006
*Pseudomonas* sp.	Polycyclic aromatic hydrocarbon polluted soil	Salicylate and phthalate pathways	Jia et al., 2008
*Arthrobacter* sp.	Polycyclic aromatic hydrocarbon -contaminated site	Phthalic pathway is more expressed than the salicylate pathway	Seo et al., 2006
*Cycloclasticus* sp. P1	Deep sea sediments	Pyrene degradation pathway	Wang et al., 2018b
*Bordetella avium* MAM-P22	Petroleum refinery wastewater	Degradation of naphthalene by production of intermediate compounds i.e., 1,2-Benzene dicarboxylic acid, Butyl-2,4-dimethyl-2-nitro-4-pentenoate, 1-Nonen-3-ol, Eicosane, Nonacosane	Abo-State et al., 2018

### Pesticides

Long-term exposure of certain microorganisms to agrochemicals can result in resistance. Therefore, they can be used in bioremediation of pesticide-contaminated sites (Khan et al., 2009b). Their performance substantially increases in the presence of specific pesticides as they use them as a nutrient and energy source (Qiu et al., 2009; Reddy et al., 2016). Their ability to degrade pesticides is essential for eliminating harmful, toxic chemicals from the environment, controlling and reducing environmental pollution (Surekha et al., 2008).

Pesticide degradation is possible through bioremediation as an alternative to conventional methods, and is a more effective, versatile, environment-friendly, and economical strategy (Finley et al., 2010). Certain microorganisms have the ability to biodegrade pesticides through specific enzymes. Enzyme catalyzed biodegradation of pesticides is a complex process that involves a series of biochemical reactions. However, the rate and final fate of different pesticides in the environment varies greatly and depends upon biotic and abiotic factors. For example, due to structure, some pesticides, such as dichlorodiphenyltrichloroethane (DDT) became more persistent and remain in the environment for longer periods than others (Kannan et al., 1994). A variety of microbes, including fungi, bacteria and actinomycetes, are able to degrade and remove pesticides from the environment (Parte et al., 2017). Bioremediation can be carried out by indigenous microflora, or through the enrichment of microbial cultures.

Bioremediation is also affected by environmental factors, such as temperature, pH, nutrients, and moisture availability. Consequently, environmental conditions should be conducive for the growth and activity of microbes for accelerated bioremediation (Vidali, 2001). The long-term application of pesticides can also promote biodegrading enzymes in the indigenous microflora, as they served as a source of carbon and energy, making the remediation of pesticide contaminated sites easier (Qiu et al., 2009). Pesticide degrading microorganisms have been isolated from a wide variety of polluted environments. Some of them have potential to degrade more than one organic pollutant. Hence, scientists are working on microbial diversity and functioning to understand their physiology, diversity, ecology, and evolution for *in situ* biotransformation of organic contaminants (Mishra et al., 2001).

As of this point, most studies have used pure cultures of microbes to observe biodegradation of pesticides. This involves the isolation of microbes from contaminated sites, their purification and characterization for degradation of different rates of specific pesticides under laboratory conditions. For example, some stains of microbes for biodegradation of DDT have been isolated from soil, sewage, animal feces, activated sludge and sediments (Johnsen, 1976; Lal and Saxena, 1982; Rochkind-Dubinsky et al., 1986). The *in situ* use of microbes for degradation of organophosphates in the pioneering work of Matsumura et al. (1968) showed that *Pseudomonas* sp. was capable of degrading dieldrin in soil in natural environments. The degradation of certain pesticides requires more than one bacterial strain. The use of this strategy is known as co-metabolism. In this process, one bacterial strain converts the original compound to an intermediate that is ultimately converted in to a final product having less or even no potential hazards for the environment. For example, the biodegradation of DDT with an alternative carbon source, which microbes used as a nutrient or energy source to transform DDT, involves co-metabolism (Bollag et al., 1990).

Neurotoxic systemic insecticides not only persist for long periods in the soil, but also are non-selective and kill beneficial insects. They are water-soluble and persist in food chains and biogeochemical cycles. There are several bacterial strains with the ability to biodegrade neonicotinoids in soil and water systems (Hussain et al., 2016). Organochloride pesticides are recalcitrant and are more resistant to biodegradation (Díaz, 2004). Bacteria are the major group among microorganisms with the potential to degrade organochloride pesticides. The degradation ability of soil bacteria from the genera *Arthrobacter, Pseudomonas, Bacillus*, and *Micrococcus* was reported by Langlois et al. (1970).

Fungi are equally important in the degradation of organochlorine pesticides. Xiao et al. (2010) conducted a study on fungal degradation of heptachlor and reported that after 14 days of incubation *P. acanthocystis, P. brevispora*, and *P. tremellosa* removed about 90, 74, 71% heptachlor, respectively. They proposed that hydrolysis and hydroxylation are the dominant reactions involved. Similarly, Ozdal et al. (2016) reported that *P. aeruginosa* G1, *Stenotrophomonas maltophilia* G2, *B. atrophaeus* G3, *Citrobacter amolonaticus* G4, and *Acinetobacter lowffii* G5 have high biodegradation ability for the organochlorine, endosulfan. Ortega-Gonzalez et al. (2015) conducted an experiment with marine fungi extracted from marine sponges. The fungi *Penicillium raistrickii, Trichoderma* sp., *Aspergillus sydowii, Penicillium miczynskii, Bionectria* sp., and *Aspergillus sydowii* were tested in solid culture medium containing 5, 10 and 15 mg of dichlorodiphenyldichloroethane (DDD), and in a liquid medium, which had the same amounts of DDD per 100 mL of liquid medium. They reported that *A. sydowii, Trichoderma* sp., and *P. miczynskii* grow well in the presence of the pesticide; however *Trichoderma* sp. performed best and was selected for further studies.

The concentration and form of the alternate carbon sources may affect the degradation potential of microbes. Fang et al. (2010) tested glucose, yeast extract, sucrose, and fructose as additional carbon sources to foster the degradation of DDTs using *Sphingobacterium* sp. and found the half-lives of DDTs significantly decreased in the presence of additional carbon sources. Glucose as an additional carbon source resulted in the fastest biodegradation of DDTs. They confirmed these *in vitro* results through field studies. Results showed a significantly lower concentration in the samples inoculated with *Sphingobacterium* sp. as compared to uninoculated control after 90 days of incubation.

Microbes play a significant role in the degradation of the nematicide oxamyl. However, little information exists regarding the types of microbes involved in its biotransformation. Rousidou et al. (2016) isolated four oxamyl-degrading bacterial strains with the potential to enhance biodegradation of oxamyl in soil. Based on the multilocus sequence analysis (MLSA), the bacterial strains belong to different subgroups of the genus *Pseudomonas*. They hydrolyzed oxamyl to oxamyl oxime, but did not use it as a carbon source, instead utilizing methylamine as source of C and N. Three of the four strains contain methylamine dehydrogenase enzyme. Furthermore, all these strains also have a gene highly homologous to a carbamate-hydrolase gene, *cehA*, which has been found in carbaryl- and carbofuran-degrading bacterial strains. A number of bacterial strains are responsible for the degradation of carbamates, such as carbofuran and carbaryl (Bano and Musarrat, 2004; Yan et al., 2007) as well as methomyl (Mohamed, 2009; Xu et al., 2009). Aldicarb is degraded by *Stenotrophomonas maltophilia* (Saptanmasi et al., 2008) and *Aminobacter* and *Mesorhizobium* spp. are also considered to be oxamyl-degrading bacterial strains (Osborn et al., 2010).

The biodegradation of organophosphorus pesticides has been extensively studied (Singh, 2008). A variety of enzymatic systems in bacteria degrades them, for example *Acinetobacter* sp., *Serratia* sp., *Proteus vulgaris*, and *Vibrio* sp. have been reported to degrade dichlorovos (Agarry et al., 2013). Malghani et al. (2009) isolated bacteria efficient in the degradation of profenofos from the genus *Pseudomonas*. Rayu et al. (2017) demonstrated that *Xanthomonas* sp. 4R3-M3 and *Pseudomonas* sp. 4H1-M3 were able to use both chlorpyriphos and 3,5,6-trichloro-2-pyridinol as a sole carbon and nitrogen source under laboratory conditions. Similarly, degradation of cyhalothrin along with other pyrethroides by *B. thuringiensis* was reported (Chen et al., 2015). Chanika et al. (2011) identified two bacterial strains, *Pseudomonas putida* and *Acinetobacter rhizosphaerae*, which were able to rapidly degrade organophosphate fenamiphos (FEN). Both strains hydrolyzed FEN to fenamiphos-phenol and ethyl-hydrogen-isopropylphosphoramidate, although it was only further transformed by *P. putida*. The two strains use FEN as a C and N source.

Due to a decrease in effectiveness of individual pesticides, mixtures containing different active ingredient groups are being developed, especially the combination of pyrethroid and organophosphorus pesticides (Moreby et al., 2001). Genetically engineered microorganism (GEM) inoculants have the potential to degrade these complex pesticides. For example, Zhang et al. (2016) succefully engineered a multifunctional *Pseudomonas putida* X3 strain by introducing methyl parathion (MP)-degrading gene. They reported that genetically engineered strain X3 is a strong bioremediation agent that showed competitive advantage in complex environment contaminated with MP and Cd. Yuanfan et al. (2010) suggested the development of GEMs could be effective for bioremediation of contamination by multiple pesticides, as indicated by GEM with the methyl parathion hydrolase gene, *mpd*, which is responsible for hydrolyzing methyl parathion to p-nitrophenol and dimethyl phosphorothioate. The development of GEMs and dual-species consortia has the potential to be used for degrading different pesticides.

Wang et al. (2018a) studied endophyte *Neurospora intermedia*, isolated from sugarcane roots grown in a diuron-treated soil. Diuron is a broad-spectrum phenylurea herbicide for pre-emergence weed control in a wide bunch of crops. Their results indicated that the strain DP8-1 was capable of degrading up to 99% diuron within 3 days under optimal degrading conditions. The degradation spectrum of DP8-1 inlcuded fenuron, monuron, metobromuron, isoproturon, chlorbromuron, linuron, and chlortoluron. Its on-site applicability, however, needs further investigation.

Microbes that can degrade more than one group of pesticides would be more efficient and economic than those with specific traits. Above all, strains with multiple plant growth promoting traits, such as the ability to solubilize zinc, promote phosphate and chitinase activity, with a high root colonization potential, and biodegrade pesticides would be most effective due to their multi-purpose applicability. As these strains can efficiently colonize the plant roots and help plant roots to proliferate, phytoremediation is more feasible and makes inoculation with these microbes an economical and applicable strategy for the remediation of pesticide-contaminated sites.

Recently, the potential of GME to degrade or accumulate contaminants is also discussed. Their impact is much wider than that of their wild relatives, improving degradation or alteration of catabolic pathways, either to protect the host plant against phytotoxicity or to improve their overall efficiency of phytoremediation. This is especially suitable when hydrophilic compounds fail to be degraded by rhizospheric microbes due to the rapid uptake by plants (Ijaz et al., 2016).

### Inorganic Pollutants

Pollution to soil, water, and air is caused by release of inorganic chemical waste by industries, auomobiles, construction companies, and fertilizers. Inorganic pollutants mainly include heavy metals which may be detoxified by using microbes in the presence or absence of plant systems. Heavy metals may be beneficial or harmful for microbes, depending upon their nature and bioavailability (Ayangbenro and Babalola, 2017). For example, some heavy metals like manganese (Mn), Fe, nickel (Ni), Mg, copper (Cu), chromium (Cr), cobalt (Co), and Zn are essential micronutrients, required in a number of physiological processes, such as forming parts of enzyme complexes, redox reactions, and the stabilization of molecules through electrostatic interactions (Bruins et al., 2000). Other heavy metals, such as arsenic (As), antimony (Sb), lead (Pb), gold (Au), cadmium (Cd), Al, silver (Ag), and mercury (Hg), are not essential, and have no biological role in the microbial body (Bruins et al., 2000). In high concentrations, they can form various complexes in microbial bodies that are highly toxic. Even essential heavy metals like Zn and Ni can also be toxic at higher concentrations.

### Microbial Detoxification of Heavy Metals

Some microbial strains develop resistance against these heavy metals and they have the ability to detoxify them. They could be a means to detoxify heavy metals at higher concentrations in the environment. Heavy metals and other ions must first enter the microbial cells for any indication of beneficial or harmful effects on microbial physiology (Nies, 1999). Many divalent heavy metals, e.g., Zn^2+^, Cu^2+^, Ni^2+^ Co^2+^, Fe^2+^, and Mn^2+^, are structurally similar, and anions of some heavy metals with oxygen resemble the anions of essential elements, for example chromate resembles sulfate, while arsenate resembles phosphate. As a result, the microbial uptake mechanisms need to be tightly controlled. Microbes use chemiosmosis, a gradient driven, very fast, and unspecific uptake systems for heavy metals (Nies, 1999) that increase their accumulation within the microbial body.

In microbial cells, toxicity may occur when heavy metals displace essential metals from their binding sites (Nies, 1999). They may also cause toxicity due to ligand interactions (Bruins et al., 2000). Heavy metals have the tendency to bind with sulfur-hydrogene (SH) groups in the microbial body and play a role in the inhibition of sensitive enzymes. The minimum concentration of heavy metals effective enough to bind with SH groups and inhibit enzyme activity is called the minimal inhibitory concentration (MIC). Some bacterial strains have an exceptionally high MIC, and therefore have a high resistance to heavy metals. For example, Yilmaz (2003) isolated and identified a bacterial strain *Bacillus circulans* EB1, with a high MIC for heavy metals. Bacterial strains with a higher MIC are preferable for bioremediation of heavy metal contaminated sites.

Other possible mechanisms of heavy metal resistance in microbes could be intra and extra-cellular sequestration, enzymatic reduction, biosorption, reduction in sensitivity of cellular targets to metal ions, and antioxidant defense system (Huang et al., 1990; Brady and Duncan, 1994; Liu et al., 2004; Xu et al., 2015). Microorganims release extracellular polymeric substances (EPS) which bind the heavy metals. Biosorption mechnisms used by EPS from *Bacillus subtilis* involve functional groups. Heavy metals, such as Cu(II) binds with anionic oxygen-bearing ligands and form inner-sphere complexes with the EPS functional groups as reported by Fang et al. (2014). These mechanisms can be useful in understanding the survival of microbes in this context.

Microbes remove heavy metal contaminants in different ways, such as biosorption, precipitation, biotransformation, bioaccumulation, complexation, enzymatic transformation of metals and phytoremediation (Liu et al., 2004; Ojuederie and Babalola, 2017; Xu et al., 2017). In bioaccumulation, microbes retain and concentrate heavy metals in their body. Bioaccumulating microbial strains can be strong candidates for decontamination of polluted soil and water as reported by Akhter et al. (2017). They isolated and identified three bacterial strains of *B. cereus*, i.e., BDBC01, AVP12, and NC7401, from rhizosphere of *Tagetes minuta* and reported that these strains have strong solubilization and accumulation potential for Cr(VI), Ni(II), and Cd(II) thus help in biosorption of these metals. Biosorption of heavy metals is the sequestration of positively charged metal ions by ionic groups on cell surfaces (Malik, 2004). Bacteria-clay mineral interactions are important in the context of metal immobilization and allocation of metals to mineral fraction. The adsorption-desorption mechanisms are affected by microbial composition and diversity, chemical behavior of metals, metal speciation and concentration, modeling method (Du et al., 2017a; Qu et al., 2017b), soil physico-chemical properties, such as clay minerals and soil pH, affecting the number of negative charge sites (Qu et al., 2017a) and plant species. The mobility of heavy metals in soils also depends upon type and concentration of ligands and sorbents, such as bacteria-mineral complexes (Du et al., 2016a). For example, citrate and humic acid enhanced Cd adsorption on *P. putida*–montmorilonite and *P. putida*–goethite composites while oxalate suppressed Cd adsorption. Phosphate ligand increased Cd sorption on *P. putida*–goethite while decreased on *P. putida*–montmorilonite composite. Recently, Qu et al. (2018) conducted a study on Pb sorption on montmorillonite-bacteria composites using a combination of atomic force microscope (AFM), X-ray diffraction (XRD), surface complexation modeling (SCM), Pb-LIII edge extended X-ray absorption fine structure (EXAFS) spectroscopy, and isothermal titration calorimetry (ITC). They observed that formation of montmorillonite, *Pseudomonas putida* complex promoted the allocation of Pb to mineral fraction and reported that SCM, EXAFS, and ITC may help in predicting the speciation and fate of Pb in soils and associated environments. For risk assessments in soils and associated environments, the heavy metal adsorption in complex systems is based on accurate modeling. In another study, Du et al. (2017a) investigated Cd adsorption on Gram-positive *Bacillus subtilis*, Gram-negative *Pseudomonas putida* and their binary mixtures with montmorillonite using SCM, Cd K-edge EXAFS, spectroscopy, and ITC. They reported that *B. subtilis* adsorbed more amounts of Cd than *P. putida* at pH < ~6 while *P. putida* was more efficient for Cd sorption to phosphate groups. This suggests that microbial composition and diversity along with biochemical behavior of trace metals are important for metal sorption in microbe-bearing environments. Qu et al. (2017a) established component additivity (CA) method with CA-site masking for Cd adsorption on goethite-*Pseudomonas putida* composites using different mass ratios. Both CA and CA-site masking models were in line with ICT data however, it was observed that CA method was excellent in simulating Cd adsorption on bacteria-iron oxides composites at low bacterial and Cd concentrations while wide deviation was observed at higher concentrations. Both models were supported by thermodynamic reaction data while these models are conditional to mineral/bacteria ratio and concentration and different models behave differently under different conditions and heavy metal concentrations (Wang et al., 2016). Moreover, interfacial complexation reations that occur between iron (hydr)oxides and bacteria should be taken into account for higher bacterial/metal concentrations.

Organic matter in complexation with iron minerals helps the adsorption of metals in soil environments. Soil microbes can further imrpove the adsorption when present in these multi-complexes. In soil environments, iron oxides make complexes with organic composites and help in the transformation trace metals. Du et al. (2017b) studied the copper adsorption on composites of synthetic goethite, cells of *Pseudomonas putida*, humic acid (HA), and their binary and ternary composites with batch adsorption experiments coupled with ITC. They reported that bacterial composites with goethite or humic acid separately and in combination help in adsorption and cycling of Cu but their affinity was less than binary composite of goethite and humic acid. Furthermore, binary and tertiary complexes of bacteria, iron oxide and humic substances affected the sequestration of heavy metals (Du et al., 2017a). During precipitation and transformation, microbes change the oxidation state of metals and metalloids to make them less harmful. The microbes used in bioremediation demonstrate a wide range of mechanisms, which change the bioavailability, transport properties, sorption characteristics and toxicity of heavy metals (Malik, 2004; Gupta et al., 2016). Metals show competitive adsorption for same type of adsorbents as they tend to bound on same types of adsorption sites on the adsorbent (Du et al., 2016b). Similarly, different microbial strains vary in their affinity for the sorption of heavy metals. For instance, fungal strain *Paecilomyces lilacinus* XLA was more efficient and eco-friendly than *Mucoromycota* sp. XLC for bioremediation of Cd^2+^ from wastewater (Xia et al., 2015).

Some studies aimed to isolate and screen metal-resistant microorganisms from polluted environments for bioremediation purposes (Pal and Paul, 2004; Abou-Shanab et al., 2007). For example, Akinbowale et al. (2007) isolated aeromonads and pseudomonads from fish, which were resistant to heavy metals. Srivastava et al. (2007) and Congeevaram et al. (2007) successfully used heavy metal resistant bacteria to detoxify heavy metal polluted sites. In the past decade, attention has been turned toward identification of bacterial strains with the potential to bioremediate polluted soils through the sequestration of toxic heavy metals and degradation of xenobiotic compounds (Braud et al., 2009; Hayat et al., 2010; Wani and Khan, 2010; Ahemad, 2012). Bioaugmentation of contaminated sites through efficient microbial strains can significantly reduce metal concentrations in polluted soil (Emenike et al., 2016; Fauziah et al., 2017). There are a number of bacterial strains belonging to the genera *Pseudomonas, Bradyrhizobium, Psychrobacter, Ochrobactrum Lysinibacillus, Rhodococcus*, and *Bacillus*, which have novel traits useful for heavy metal decontamination from polluted environments (Dary et al., 2010; Ma et al., 2010; Wani and Khan, 2010; Emenike et al., 2016). Fungi are also equally important in the remediation of metal polluted sites. For example, Xu et al. (2017) reported that *Paecilomyces lilacinus* XLA has the ability to reduce Cr^6+^ that can reduce over 90% of Cr^6+^ in growth media with Cr^6+^ concentration below 100 mg L^−1^ at pH 6 after 14 days of incubation. They reported that XLA used biosorption, biotransformation, and bioaccumulation as the major mechanisms for reduction of chromium. The XLA might have detoxified the reactive oxygen species produced by external Cr^6+^ through its efficient antioxidant enzyme system. The efficiency of these fungal species can be affected by soil conditions, such as pollution level of metals, pH, EC, temperature, and nutrient status of soils.

### Phytoremediation of Heavy Metals

Phytoremediation is based on hyper-accumulating plant species. The phytoremediation ability of plants depends on environmental conditions, the quantity of heavy metals present at the site, soil type, and microbial number and diversity (Ojuederie and Babalola, 2017). To accelerate the process, scientists are exploring plant–microbe interactions, combining the capabilities of rhizosphere bacteria to improve metal uptake by the plant. Kuffner et al. (2008) isolated ten rhizosphere isolates obtained from heavy metal accumulating willows (*Salix* sp.). They belonged to the genera *Agromyces, Flavobacterium, Serratia, Pseudomonas*, and *Streptomyces*. Plant growth, as well as Zn and Cd uptake potential of these strains were measured. The strain *Agromyces* AR33 almost doubled Zn and Cd extractability, which was attributed to the improvement of release of Zn and Cd specific ligands. Some other strains were helpful in improving Zn and Cd uptake of *Salix caprea* plantlets. However, they might have used different plant-microbe interactions to improve heavy metal uptake, except IAA production, ACC deaminase activity and siderophore production.

Some PGPR have the ability to improve heavy metal uptake in crops. For example, Ghasemi et al. (2018) conducted an experiment to study the effectiveness of five bacterial isolates with plant growth promoting traits on improving growth, health and Ni phytoextraction capacity of three Ni-hyperaccumulators, *Odontarrhena inflate, O. bracteata*, and *O. serpyllifolia*. Plants were inoculated with five rhizobacterial strains (previously isolated from *O. serpyllifolia*) and grown for 3 months. The bacterial inoculants enhanced Ni removal due to the stimulation in growth and/or increase in shoot Ni concentration, but the effectiveness of these strains varied with bacterial strain, plant species and soil type (Ghasemi et al., 2018).

Bacteria with ACC deaminase can potentially induce heavy metal stress tolerance in crop plants. Consequently, they could enhance the phytoextraction and phytoremediation potential of plants. Four bacterial strains from Ni-contaminated soils were isolated by Rodriguez et al. (2008) on the basis of ACC deaminase activity. They were identified as *Pseudomonas putida* Biovar B, and also have plant growth promoting traits, such as indole acetic acid and siderophores production, in addition to ACC deaminase activity. They were tolerant of up to 13.2 mM Ni in a culture medium. Based its effectiveness from the laboratory results, strain HS-2 was tested in pot experiments. It was observed that canola plants inoculated with HS-2 strain accumulated more biomass and had higher Ni contents in shoots and roots. According to these results, HS-2 could be a potential inoculant for the phytoremediation of Ni contaminated sites (Rodriguez et al., 2008).

Endophytic bacteria have also been shown to improve heavy metal stress tolerance in crop plants. For example, Sheng et al. (2008) isolated two Pb resistant endophytic bacteria, *Pseudomonas fluorescens* G10 and *Microbacterium* sp. G16, from the roots of canola plants grown in Pb contaminated fields. The isolated strains were resistant to heavy metals and successfully improved growth of canola plants in pot experiment. Zhang et al. (2011b) characterized three ACC deaminase producing endophytic bacteria, *Ralstonia* sp. J1-22-2, *Pantoea agglomerans* Jp3-3, and *Pseudomonas thivervalensis* Y1-3-9 out of 100 isolates isolated from copper-tolerant plants. They reported that these bacteria promoted plant growth and copper (Cu) accumulation in canola plants in a pot experiment This supports the endophytic bacterial-assisted phytoremediation strategy for Cu-contaminated environments.

As seen in the above studies, microbes can be part of an innovative strategy to remediate heavy metal contaminated soils. Under varying conditions and in different crops, bacterial strains showed the ability to improve plant growth, such as IAA production, ACC deaminase activity, siderophores production, and heavy metal uptake, through bioaccumulation, biotransformation, precipitation, and biosorption. However, there are still gaps in the understanding of specific plant-microbe interactions involved in the bioremediation of metal contaminated sites. There are bacterial strains with no IAA and siderospores production ability, and no ACC deaminase activity, which have enhanced metal uptake and accumulation in plant organs. Bioremediation technologies are necessary for the detoxification of metal-contaminants in polluted environments (Emenike et al., 2018a,b), to prevent their toxic effects on the environment and living organisms.

## Conclusion and Future Prospects

The efforts to feed a burgeoning population by increasing yields with new crop varieties and agrochemicals have significantly violated global ecosystems. Moreover, industrialization and urbanization have put extra pressure on soil and water resources around the cities and towns, engulfing fertile agricultural lands. Effluents and exhaust from industries and automobiles pollute soil, water, and atmosphere, add contaminants into food chain, and create unhealthy conditions for human life.

This paper overviewed methods to restore and sustain the environment with the use of microorganisms for site decontamination. It demonstrated that microbes are effective in the degradation of agrochemicals, industrial effluents, and petroleum products. Microorganisms have great potential to decontaminate polluted sites though their direct role in the degradation of organic pollutants and detoxification of inorganic compounds, and their indirect role of decreasing the need for agrochemicals through plant growth promoting mechanisms.

The reviewed literature shows that microbial inoculants can be successfully used as biofertilizers and biopesticides by using diverse plant growth promoting traits. Microorganisms either improve plant growth by direct effects, such as BNF, hormone production, nutrients solubilization, or are indirectly involved in the protection of plants from biotic and abiotic stresses. Other mechanisms are antibiotic production for the suppression of phytodiseases, chitinase and catalase activities for the degradation of fungal cell wall, exopolysaccharides and siderophore production to make nutrients unavailable for disease causing organisms, and ACC deaminase activity to reduce the negative impact of stressed environments. Through these and other still unknown mechanisms, microbes improve plant growth and productivity, without fertilizers and pesticides. Most of the studies conducted so far have focused on the use of microbial inoculants for agricultural productivity. As these microbes may also affect ecology and soil microbial community structure, leading to improved soil health, future research should be focused on quantifying the impact of microbial inoculants on ecosystem and soil health.

This review also outlines the potential for microbial inoculants in bioremediation and detoxification of pollutants from the environment. Microbes from different genera of rhizobacteria, endophytes, and fungi have been identified for their ability to degrade organic pollutants and detoxify heavy metals. They are equally effective in degrading pesticides, azodyes, and polyaromatic hydrocarbons, along with the detoxification of heavy metals from industrial waste. Studies on consortial inoculants should be the priority for the degradation of complex agrochemicals, polyaromatic hydrocarbons, and azodyes. Moreover, root colonization efficiency of these microbes should also be further studied to increase their effectiveness as bioremediators specific plant-microbe interactions in the decontamination of environmental pollutants need to be explored, as it has been suggested that microbes use unknown mechanisms to enhance metal uptake and accumulation in plants. Research is also needed to find out the pathways for the degradation of industrial effluents by microbial strains

The use of genetically engineered microbes has also been reported in literature. Comprehensive research is needed as little is known about these microbes *in situ*. Their behavior in a natural environment and their impact on soil health and soil microbial community structure and functional genes should be studied extensively. Furthermore, the specific mechanisms and genes involved for bioremediation and detoxification of pollutants should also be explored. There is a need to investigate site-specific microbial communities under a wide range of environmental conditions. Another area of interest is the formulation of suitable inoculants and the testing of their environmental impact. Only the tip of the iceberg has been identified, while the vast majority of beneficial species and their potential have yet to be unraveled.

## Author Contributions

MA developed the idea, prepared initial structure, coordinated with co-authors throughout the manuscript development and finalized the submission accordingly. LP helped in the preparation of manuscript for the sections biofertilizers and biopesticides and finalized the bibliography. TH guided throughout the preparation of the manuscript and in the write-up of the section heavy metal impact on environment, soil fertility and bioremediation of heavy metals. ZZ provided data on use of microbes as biofertilizers and biopesticides and degradation of organic pollutants, and performed final editing of the manuscript before submission. AH collected review on detoxification of industrial effluents by microbes and prepared the draft on the section. FR, RS, and SS edited the manuscript and guided throughout the preparation of manuscript.

### Conflict of Interest Statement

The authors declare that the research was conducted in the absence of any commercial or financial relationships that could be construed as a potential conflict of interest.

## References

[B1] Abd El-Rahim W. M. Zaki E. A. (2005). Functional and molecular characterization of native Egyptian fungi capable of removing textile dyes. Arab J. Biotechnol. 8, 189–200.

[B2] Abo-State M. A. M. Riad B. Y. Bakr A. A. Abdel Aziz M. F. (2018). Biodegradation of naphthalene by *Bordetella avium* isolated from petroleum refinery wastewater in Egypt and its pathway. J. Rad. Res. Appl. Sci. 11, 1–9. 10.1016/j.jrras.2017.10.001

[B3] Abou-Shanab R. A. Van Berkum P. Angle J. S. (2007). Heavy metal resistance and genotypic analysis of metal resistance genes in gram-positive and gram-negative bacteria present in Ni-rich serpentine soil and in the rhizosphere of *Alyssum murale*. Chemosphere 68, 360–367. 10.1016/j.chemosphere.2006.12.051 17276484

[B4] Adelana S. O. Adeosun T. A. Adesina A. O. Ojuroye M. O. (2011). Environmental pollution and remediation: challenges and management of oil spillage in the Nigerian coastal areas. Am. J. Sci. Ind. Res. 2, 834–845. 10.5251/ajsir.2011.2.6.834.845

[B5] Agarry S. E. Olu-Arotiowa O. A. Aremu M. O. Jimoda L. A. (2013). Biodegradation of dichlorovos (organophosphate pesticide) in soil by bacterial isolates. J. Nat. Sci. Res. 3, 12–16.

[B6] Ahamd M. Zeshan M. S. H. Nasim M. Zahir Z. A. Nadeem S. M. Nazli F. (2015). Improving the productivity of cucumber through combined application of organic fertilizers and *Pseudomonas fluorescens*. Pak. J. Agri. Sci. 52, 1011–1016.

[B7] Ahemad M. (2012). Implications of bacterial resistance against heavy metals in bioremediation: a review. IIOAB J. 3, 39–46.

[B8] Ahemad M. Kibret M. (2014). Mechanisms and applications of plant growth promoting rhizobacteria: current perspective. J. King Saud Univ. Sci. 26, 1–20. 10.1016/j.jksus.2013.05.001

[B9] Ahmad M. Jamil M. Zahir Z. A. Nadeem S. M. Kharal M. A. Saeed A. (2016). Effectiveness of *Pseudomonas fluorescens* and L-tryptophan to improve seedlings growth of onion (*Alium cepa* L.). Soil Environ. 35, 85–90.

[B10] Ahmad M. Zahir Z. A. Asghar H. N. Arshad M. (2012). The combined application of rhizobial strains and plant growth promoting rhizobacteria improves growth and productivity of mung bean (*Vigna radiata* L.) under salt-stressed conditions. Ann. Microbiol. 62, 1321–1330. 10.1007/s13213-011-0380-9

[B11] Ahmad M. Zahir Z. A. Asghar H. N. Asghar M. (2011). Inducing salt tolerance in mung bean through coinoculation with rhizobia and plant-growth-promoting rhizobacteria containing 1-aminocyclopropane-1-carboxylate deaminase. Can. J. Microbiol. 57, 578–589. 10.1139/w11-044 21770816

[B12] Ahmad M. Zahir Z. A. Khalid M. Nazli F. Arshad M. (2013a). Efficacy of *Rhizobium* and *Pseudomonas* strains to improve physiology, ionic balance and quality of mung bean under salt-affected conditions on farmer's fields. Plant Physiol. Biochem. 63, 170–176. 10.1016/j.plaphy.2012.11.024 23262185

[B13] Ahmad M. Zahir Z. A. Nadeem S. M. Nazli F. Jamil M. Khalid M. (2013b). Field evaluation of *Rhizobium* and *Pseudomonas* strains to improve growth, nodulation and yield of mung bean under salt-affected conditions. Soil Environ. 32, 158–166.

[B14] Ahmad M. Zahir Z. A. Nazli F. Akram F. Arshad M. Khalid M. (2013c). Effectiveness of halo-tolerant, auxin producing *Pseudomonas* and *Rhizobium* strains to improve osmotic stress tolerance in mung bean (*Vigna radiata* L.). Braz. J. Microbiol. 44, 1341–1348. 10.1590/S1517-83822013000400045 24688532 PMC3958208

[B15] Ahmadi K. Razavi B. S. Maharjan M. Kuzyakov Y. Kostka S. J. Carminati A. (2018). Effects of rhizosphere wettability on microbial biomass, enzyme activities and localization. Rhizosphere 7, 35–42. 10.1016/j.rhisph.2018.06.010

[B16] Akhter K. Ghous T. Andleeb S. Nasim F. H. Ejaz S. Zain-ul-Abdin (2017). Bioaccumulation of heavy metals by metal-resistant bacteria isolated from tagetes minuta rhizosphere, growing in soil adjoining automobile workshops. Pak. J. Zool. 49, 1841–1846. 10.17582/journal.pjz/2017.49.5.1841.1846

[B17] Akinbowale O. L. Peng H. Grant P. Barton M. D. (2007). Antibiotic and heavy metal resistance in motile aeromonads and pseudomonads from rainbow trout (*Oncorhynchus mykiss*) farms in Australia. Int. J. Antimicrob. Agents 30, 177–182. 10.1016/j.ijantimicag.2007.03.012 17524624

[B18] Aktar M. W. Sengupta D. Chowdhury A. (2009). Impact of pesticides use in agriculture: their benefits and hazards. Interdiscip. Toxicol. 2, 1–12. 10.2478/v10102-009-0001-7 21217838 PMC2984095

[B19] Ali H. (2010). Biodegradation of synthetic dyes–a review. Water Air Soil Pollut. 213, 251–273. 10.1007/s11270-010-0382-4

[B20] Anderson B. Phillips B. Hunt J. Siegler K. Voorhees J. Smalling K. (2014). Impacts of pesticides in a Central California estuary. Environ. Monit. Assess. 185, 1801–1814. 10.1007/s10661-013-3494-7 24464329

[B21] Antil R. S. Dinesh Dahiya S. S. (2007). Utilization of sewer water and its significance in INM, in Proceedings of ICAR sponsored Winter School on Integrated Nutrient Management, Department of Soil Science and Directorate of Human Resource Management, CCS Haryana Agricultural University (Hisar, India), Dec. 4–24, 2007, 79–83.

[B22] Anwar Y. Hanafy A. A. E. Sabir J. S. Al-Garni S. M. Ahmed M. M. M. (2016). Microbes using PAHs as energy source: relationship with diseases. Res. J. Biotechnol. 11, 94–109.

[B23] Argaw A. (2018). Integrating inorganic NP application and *Bradyrhizobium* inoculation to minimize production cost of peanut (*Arachis hypogea* L.) in eastern Ethiopia. Agric. Food Secur. 7:20 10.1186/s40066-018-0169-1

[B24] Arslan-Alaton I. (2007). Degradation of a commercial textile biocide with advanced oxidation processes and ozone. J. Environ. Manage. 82, 145–154. 10.1016/j.jenvman.2005.12.021 16624477

[B25] Ayangbenro A. S. Babalola O. O. (2017). A new strategy for heavy metal polluted environments: a review of microbial biosorbents. Int. J. Environ. Res. Public Health. 14:94. 10.3390/ijerph14010094 28106848 PMC5295344

[B26] Aydin S. KaraCay H. A. Shahi A. Gokce S. Ince B. Ince O. (2016). Aerobic and anaerobic fungal metabolism and omics insights for increasing polyaromatic hydrocarbons biodegradation. Fungal Biol. Rev. 31, 61–72. 10.1016/j.fbr.2016.12.001

[B27] Babu B. R. Parande A. K. Kumar S. A. Bhanu S. U. (2011). Treatment of dye effluent by electrochemical and biological processes. Open J. Saf. Sci. Technol. 1, 12–18. 10.4236/ojsst.2011.11002

[B28] Bai Z. G. Dent D. L. Olsson L. Schaepman M. E. (2008). Proxy global assessment of land degradation. Soil Use Manage. 24, 223–234. 10.1111/j.1475-2743.2008.00169.x

[B29] Bailey A. Chandler D. Grant W. P. Greaves J. Prince G. Tatchell M. (2010). Biopesticides: Pest Management and Regulation. Oxfordshire: CABI.

[B30] Bandi S. Sivasubramanian P. (2012). Management of *Thrips tabaci* Lindeman in onion using *Pseudomonas fluorescens* Migula through induced resistance. J. Biopest. 5, 1–3.

[B31] Bano N. Musarrat J. (2004). Characterization of a novel carbofuran degrading *Pseudomonas* sp. with collateral biocontrol and plant growth promoting potential. FEMS Microbiol. Lett. 231, 13–17. 10.1016/S0378-1097(03)00894-2 14769460

[B32] Barnawal D. Bharti N. Pandey S. S. Pandey A. Chanotiya C. S. Kalra A. (2017). Plant growth-promoting rhizobacteria enhance wheat salt and drought stress tolerance by altering endogenous phytohormone levels and TaCTR1/TaDREB2 expression. Physiol. Planta. 161, 502–514. 10.1111/ppl.12614 28786221

[B33] Bashan Y. Levanony H. (1990). Current status of *Azospirillum* inoculation technology: *Azospirillum* as a challenge for agriculture. Can. J. Microbiol. 36, 591–608. 10.1139/m90-105

[B34] Beneduzi A. Moreira F. Costa P. B. Vargas L. K. Lisboa B. B. Favreto R. (2013). Diversity and plant growth promoting evaluation abilities of bacteria isolated from sugarcane cultivated in the South of Brazil. Appl. Soil Ecol. 63, 94–104. 10.1016/j.apsoil.2012.08.010

[B35] Bhattacharyya P. N. Jha D. K. (2012). Plant growth-promoting rhizobacteria (PGPR): emergence in agriculture. World J. Microbiol. Biotechnol. 28, 1327–1350. 10.1007/s11274-011-0979-9 22805914

[B36] Bollag J. M. Liu S. Y. Cheng H. H. (1990). Biological transformation processes of pesticides, in Pesticides in the Soil Environment: Processes, Impacts, and Modeling, ed H. H. Cheng (Madison, WI: Soil Science Society of America), 169–211.

[B37] Brady D. Duncan J. R. (1994). Bioaccumulation of metal cations by *Saccharomyces cerevisiae*. Appl. Microbiol. Biotechnol. 41, 149–154. 10.1007/BF00166098

[B38] Braud A. Jézéquel K. Bazot S. Lebeau T. (2009). Enhanced phytoextraction of an agricultural Cr and Pb contaminated soil by bioaugmentation with siderophore-producing bacteria. Chemosphere 74, 280–286. 10.1016/j.chemosphere.2008.09.013 18945474

[B39] Bruins M. R. Kapil S. Oehme F. W. (2000). Microbial resistance to metals in the environment. Ecotoxicol. Environ. Saf. 45, 198–207. 10.1006/eesa.1999.1860 10702338

[B40] Camerini S. Senatore B. Lonardo E. Imperlini E. Bianco C. Moschetti G.. (2008). Introduction of a novel pathway for IAA biosynthesis to rhizobia alters vetch root nodule development. Arch. Microbiol. 190, 67–77. 10.1007/s00203-008-0365-7 18415080

[B41] Carpenter S. R. Mooney H. A. Agard J. Capistrano D. Defries R. S. Díaz S.. (2009). Science for managing ecosystem services: beyond the millennium ecosystem assessment. Proc. Natl. Acad. Sci. U.S.A. 106, 1305–1312. 10.1073/pnas.0808772106 19179280 PMC2635788

[B42] Chamani H. E. Yasari E. Pirdashti H. (2015). Response of yield and yield components of rice (*Oryza sativa* L. cv. Shiroodi) to different phosphate solubilizing microorganisms and mineral phosphorous. Int. J. BioSci. 6, 70–75.

[B43] Chambers P. A. Guy M. Roberts E. S. Charlton M. N. Kent R. Gagnon C. (2001). Nutrients and Their Impact on the Canadian Environment. Agriculture and Agri-Food Canada, Environment Canada, Fisheries and Oceans Canada. Health Canada and Natural Resources Canada. Hull, QC: Minister of Public Works and Government Services Canada, 241p.

[B44] Chanika E. Georgiadou D. Soueref E. Karas P. Karanasios E. Tsiropoulos N. G.. (2011). Isolation of soil bacteria able to hydrolyze both organophosphate and carbamate pesticides. Bioresour. Technol. 102, 3184–3192. 10.1016/j.biortech.2010.10.145 21112209

[B45] Chen H. (2006). Recent advances in azo dye degrading enzyme research. Curr. Protein Pept. Sci. 7, 101–111. 10.2174/138920306776359786 16611136 PMC5863238

[B46] Chen S. Deng Y. Chang C. Lee J. Cheng Y. Cui Z.. (2015). Pathway and kinetics of cyhalothrin biodegradation by *Bacillus thuringiensis* strain ZS-19. Sci. Rep. 5:8784. 10.1038/srep08784 25740758 PMC4350101

[B47] Chen S. Peng J. Duan G. (2016). Enrichment of functional microbes and genes during pyrene degradation in two different soils. J. Soils Sediments 16, 417–426. 10.1007/s11368-015-1204-5

[B48] Compant S. Duffy B. Nowak J. Clément C. AitBarka E. (2005). Use of plant growth-promoting bacteria for biocontrol of plant diseases: principles, mechanisms of action, and future prospects. Appl. Environ. Microbiol. 71, 4951–4959. 10.1128/AEM.71.9.4951-4959.2005 16151072 PMC1214602

[B49] Congeevaram S. Dhanarani S. Park J. Dexilin M. Thamaraiselvi K. (2007). Biosorption of chromium and nickel by heavy metal resistant fungal and bacterial isolates. J. Hazard. Mater. 146, 270–277. 10.1016/j.jhazmat.2006.12.017 17218056

[B50] Dai Z.-C. Fu W. Wan L.-Y. Cai H.-H. Wang N. Qi S.-S.. (2016). Different growth promoting effects of endophytic bacteria on invasive and native clonal plants. Front. Plant Sci. 7:706. 10.3389/fpls.2016.00706 27252722 PMC4878316

[B51] Dandie C. E. Thomas S. M. Bentham R. H. McClure N. C. (2004). Physiological characterization of *Mycobacterium* sp. strain 1B isolated from a bacterial culture able to degrade high-molecular-weight polycyclic aromatic hydrocarbons. J. Appl. Microbiol. 97, 246–255. 10.1111/j.1365-2672.2004.02087.x 15239690

[B52] Dary M. Chamber-Pérez M. A. Palomares A. J. Pajuelo E. (2010). “*In situ*” phytostabilisation of heavy metal polluted soils using *Lupinus luteus* inoculated with metal resistant plant-growth promoting rhizobacteria. J. Hazard. Mater. 177, 323–330. 10.1016/j.jhazmat.2009.12.035 20056325

[B53] Dean D. R. Jacobson M. R. (1992). Biochemical genetics of nitrogenase, in Biological Nitrogen Fixation, eds Stacey G. Burris R. H. Evans H. J. (New York, NY: Chapman and Hall), 763–834.

[B54] Díaz E. (2004). Bacterial degradation of aromatic pollutants: a paradigm of metabolic versatility. Int. Microbiol. 7, 173–180. 15492931

[B55] Dodor D. E. Tabatabai M. A. (2003). Effect of cropping systems on phosphatases in soils. J. Plant Nutr. Soil Sci. 166, 7–13. 10.1002/jpln.200390016

[B56] Dresback K. Ghoshal D. Goyal A. (2001). Phycoremediation of trichloroethylene (TCE). Physiol. Mol. Biol. Plants 7, 117–123.

[B57] Du H. Chen W. Cai P. Rong X. Chen C. Huang Q. (2016a). Cadmium adsorption on bacteria–mineral mixtures: effect of naturally occurring ligands. Eur. J. Soil Sci. 67, 641–649. 10.1111/ejss.12373

[B58] Du H. Chen W. Cai P. Rong X. Huang Q. (2016b). Competitive adsorption of Pb and Cd on bacteria–montmorillonite composite. Environ. Poll. 218, 168–175. 10.1016/j.envpol.2016.08.022 27566847

[B59] Du H. Lin Y. Chen W. Cai P. Rong X. Shi Z. (2017b). Copper adsorption on composites of goethite, cells of *Pseudomonas putida* and humic acid. Eur. J. Soil Sci. 68, 514–523. 10.1111/ejss.12430

[B60] Du H. Qu C. Liu J. Chen W. Cai P. Shi Z.. (2017a). Molecular investigation on the binding of Cd(II) by the binary mixtures of montmorillonite with two bacterial species. Environ. Poll. 229, 871–878. 10.1016/j.envpol.2017.07.052 28754562

[B61] Edwards M. Topp E. Metcalfe C. D. Li H. Gottschall N. Bolton P.. (2009). Pharmaceutical and personal care products in tile drainage following surface spreading and injection of dewatered municipal biosolids to an agricultural field. Sci. Total Environ. 407, 4220–4230. 10.1016/j.scitotenv.2009.02.028 19394680

[B62] Ellis E. C. Klein Goldewijk K. Siebert S. Lightman D. Ramankutty N. (2010). Anthropogenic transformation of the biomes, 1700 to 2000. Global Ecol. Biogeogr. 19, 589–606. 10.1111/j.1466-8238.2010.00540.x

[B63] Emenike C. U. Agamuthu P. Fauziah S. H. (2016). Blending *Bacillus* sp., *Lysinibacillus* sp., and *Rhodococcus* sp. for optimal reduction of heavy metals in leachate contaminated soil. Environ. Earth Sci. 75:26 10.1007/s12665-015-4805-9

[B64] Emenike C. U. Jayanthi B. Agamuthu P. Fauziah S. H. (2018a). Biotransformation and removal of heavy metals: a review of phytoremediation and microbial remediation assessment on contaminated soil. Environ. Rev. 26, 156–168. 10.1139/er-2017-0045

[B65] Emenike C. U. Liew W. Fahmi M. G. Jalil K. N. Pariathamby A. Hamid F. S. (2018b). Optimal removal of heavy metals from leachate contaminated soil using bioaugmentation process. Clean Soil Air Water 45:1500802 10.1002/clen.201500802

[B66] EPA (2016). Greenhouse Gas Emissions, US Environmental Protection Agency. Available online at: https://www.epa.gov/ghgemissions/overview-greenhouse-gases/ (Accessed November 24, 2016).

[B67] Etminani F. Harighi B. (2018). Isolation and identification of endophytic bacteria with plant growth promoting activity and biocontrol potential from wild pistachio trees. Plant Pathol. J. 34, 208–217. 10.5423/PPJ.OA.07.2017.0158 29887777 PMC5985647

[B68] Fang H. Dong B. Yan H. Tang F. Yu Y. (2010). Characterization of a bacterial strain capable of degrading DDT congeners and its use in bioremediation of contaminated soil. J. Hazard. Mater. 184, 281–289. 10.1016/j.jhazmat.2010.08.034 20828928

[B69] Fang L. Yang S. Huang Q. Xue A. Cai P. (2014). Biosorption mechanisms of Cu(II) by extracellular polymeric substances from *Bacillus subtilis*. Chem. Geol. 386, 143–151. 10.1016/j.chemgeo.2014.08.017

[B70] FAO (2013). Food and Agriculture Organization of the United Nations, Statistics Division. Available online at: http://www.fao.org/statistics/databases/en/ (Accessed November 24, 2016).

[B71] Fauziah S. H. Agamuthu P. Hashim R. Izyani A. K. Emenike C. U. (2017). Assessing the bioaugmentation potentials of individual isolates from landfill on metal-polluted soil. Environ. Earth Sci. 76:401 10.1007/s12665-017-6739-x

[B72] Finley S. D. Broadbelt L. J. Hatzimanikatis V. (2010). *In silico* feasibility of novel biodegradation pathways for 1,2,4-trichlorobenzene. BMC Systems Biol. 4:7. 10.1186/1752-0509-4-7 20122273 PMC2830930

[B73] Fituma T. Tamado T. Anteneh A. (2018). Effect of inoculating *Bradyrhizobium* on phosphorus use efficiency and nutrient uptake of soybean intercropped with sugarcane in calcareous soil of metahara, Central Rift Valley, Ethiopia. Adv. Crop Sci. Tech. 28, 17–32. 10.4172/2329-8863.1000290

[B74] Fosso-Kankeu E. Mulaba-Bafubiandi A. F. Mamba B. B. Barnard T. G. (2011). Prediction of metal-adsorption behaviour in the remediation of water contamination using indigenous microorganisms. J. Environ. Manage. 92, 2786–2793. 10.1016/j.jenvman.2011.06.025 21737198

[B75] Frankowski J. Lorito M. Scala F. Schmid R. Berg G. Bahl H. (2001). Purification and properties of two chitinolytic enzymes of *Serratia plymuthica* HRO-C48. Arch. Microbiol. 176, 421–426. 10.1007/s002030100347 11734885

[B76] Fredslund L. Sniegowski K. Wick L. Y. Jacobsen C. S. De Mot R. Springael D. (2008). Surface motility of polycyclic aromatic hydrocarbon (PAH)-degrading mycobacteria. Res. Microbiol. 159, 255–262. 10.1016/j.resmic.2008.02.007 18440203

[B77] Frische T. Egerer S. Matezki S. Pickl C. Wogram J. (2018). 5-Point programme for sustainable plant protection. Environ. Sci. Eur. 30:8. 10.1186/s12302-018-0136-2 29576997 PMC5849641

[B78] Fulekar M. H. (2017). Microbial degradation of petrochemical waste-polycyclic aromatic hydrocarbons. Bioresour. Bioprocess. 4, 1–16. 10.1186/s40643-017-0158-4 28725525 PMC5493705

[B79] Gadd G. M. Sayer G. M. (2000). Fungal transformations of metals and metalloids, in Environmental Microbe-Metal Interactions, ed Lovley D. R. (Washington, DC: American Society for Microbiology), 237–256.

[B80] Galazka A. Gawryjolek K. Frac J. G. Ksjezak J. (2017). Microbial community diversity and the interaction of soil under maize growth in different cultivation techniques. Plant Soil Environ. 63, 264–270. 10.17221/171/2017-PSE

[B81] Gałazka A. Grzadziel J. (2018). Fungal genetics and functional diversity of microbial communities in the soil under long-term monoculture of maize using different cultivation techniques. Front. Microbiol. 9:76. 10.3389/fmicb.2018.00076 29441054 PMC5797640

[B82] Galazka A. Grzadziel J. Galazka R. Ukalska-Jaruga A. Strzelecka J. Smreczak B. (2018). Genetic and functional diversity of bacterial microbiome in soils with long term impacts of petroleum hydrocarbons. Front. Microbiol. 22:1923 10.3389/fmicb.2018.01923 PMC6113661 30186255

[B83] Galindo F. S. Filho T. M. C. Salatier B. Ludkiewicz M. G. Z. Rosa P. A. L. Tritapepe C. A. (2018). Technical and economic viability of co-inoculation with *Azospirillum brasilense* in soybean cultivars in the Cerrado. Rev. Bras. Eng. Agríc. Ambient. 22, 51–56. 10.1590/1807-1929/agriambi.v22n1p51-56

[B84] Gamalero E. Glick B. R. (2011). Mechanisms used by plant growth-promoting bacteria, in Bacteria in Agrobiology: Plant Nutrient Management, ed Maheshwari D. K. (Berlin; Heidelberg: Springer), 17–46.

[B85] Gandhi A. Muralidharan G. (2016). Assessment of zinc solubilizing potentiality of *Acinetobacter* sp. isolated from rice rhizosphere. Eur. J. Soil Biol. 76, 1–8. 10.1016/j.ejsobi.2016.06.006

[B86] Gao Y. Liu Q. Zang P. Li X. Ji Q. He Z. (2015). An endophytic bacterium isolated from Panax ginseng CA Meyer enhances growth, reduces morbidity, and stimulates ginsenoside biosynthesis. Phytochem. Lett. 11, 132–138. 10.1016/j.phytol.2014.12.007

[B87] Garcia D. K. (2012). Symphony of the Soil. Lily Films, USA. Available online at: http://www.symphonyofthesoil.com/the-films/symphony-of-the-soil/ (Accessed November 24, 2016).

[B88] García J. E. Maroniche G. Creus C. Suárez-Rodríguez R. Ramirez-Trujillo J. A. Groppa M. D. (2017). *In vitro* PGPR properties and osmotic tolerance of different *Azospirillum* native strains and their effects on growth of maize under drought stress. Microbiol. Res. 202, 21–29. 10.1016/j.micres.2017.04.007 28647119

[B89] Ghasemi Z. Ghaderian S. M. Rodríguez-Garrido B. Prieto-Fernández A. Kidd P. S. (2018). Plant species-specificity and effects of bioinoculants and fertilization on plant performance for nickel phytomining. Plant Soil 425, 265–285. 10.1007/s11104-017-3553-x

[B90] Ghorchiani M. Etesami H. Alikhani H. A. (2018). Improvement of growth and yield of maize under water stress by co-inoculating an arbuscular mycorrhizal fungus and a plant growth promoting rhizobacterium together with phosphate fertilizers. Agric. Ecosys. Environ. 258, 59–70. 10.1016/j.agee.2018.02.016

[B91] Giordano W. Hirsch A. M. (2004). The expression of *MaEXP1*, a *Melilotus alba* expansin gene, is upregulated during the sweet clover-*Sinorhizobium meliloti* interaction. MPMI 17, 613–622. 10.1094/MPMI.2004.17.6.613 15195944

[B92] Glick B. R. (2012). Plant growth promoting bacteria: mechanisms and applications. Scientifica 2012, 1–15. 10.6064/2012/963401 24278762 PMC3820493

[B93] Glick B. R. Penrose D. M. Li J (1998). A model for the lowering of plant ethylene concentrations by plant growth promoting rhizobacteria. J. Theor. Biol. 190, 63–68. 10.1006/jtbi.1997.0532 9473391

[B94] Goldstein A. H. (2000). Bioprocessing of rock phosphate ore: essential technical considerations for the development of a successful commercial technology, in Proceedings of the 4th International Fertilizer Association Technical Conference (Paris: IFA) (Vol. 220).

[B95] González M. B. R. Lopez J. G. (2013). Beneficial Plant-Microbial Interactions: Ecology and Applications. New York, NY: CRC Press.

[B96] Gottschall N. Edwards M. Topp E. Bolton P. Payne M. Curnoe W. E.. (2009). Nitrogen, phosphorus, and bacteria tile and groundwater quality following direct injection of dewatered municipal biosolids into soil. J. Environ. Qual. 38, 1066–1075. 10.2134/jeq2008.0085 19329694

[B97] Gouda S. Kerry R. G. Das G. Paramithiotis S. Shin H. S. Patra J. K. (2018). Revitalization of plant growth promoting rhizobacteria for sustainable development in agriculture. Microbiol. Res. 206, 131–140. 10.1016/j.micres.2017.08.016 29146250

[B98] Gupta A. Joia J. Sood A. Sood R. Sidhu C. Kaur G. (2016). Microbes as potential tool for remediation of heavy metals: a review. J. Microb. Biochem. Technol. 8, 364–372. 10.4172/1948-5948.1000310

[B99] Haas D. Keel C. (2003). Regulation of antibiotic production in root-colonizing *Pseudomonas* spp. and relevance for biological control of plant disease. Annu. Rev. Phytopathol. 41, 117–153. 10.1146/annurev.phyto.41.052002.095656 12730389

[B100] Harding D. P. Raizada M. N. (2015). Controlling weeds with fungi, bacteria and viruses: a review. Front. Plant Sci. 6:659. 10.3389/fpls.2015.00659 26379687 PMC4551831

[B101] Hayat R. Ali S. Amara U. Khalid R. Ahmed I. (2010). Soil beneficial bacteria and their role in plant growth promotion: a review. Ann. Microbiol. 60, 579–598. 10.1007/s13213-010-0117-1

[B102] He Z. L. Bian W. Zhu J. (2002). Screening and identification of microorganisms capable of utilizing phosphate adsorbed by goethite. Commun. Soil Sci. Plant Anal. 33, 647–663. 10.1081/CSS-120003057

[B103] Hennessee C. T. Li Q. X. (2016). Effects of polycyclic aromatic hydrocarbon mixtures on degradation, gene expression, and metabolite production in four *Mycobacterium* species. Appl. Environ. Microbiol. 82, 3357–3369. 10.1128/AEM.00100-16 27037123 PMC4959237

[B104] Henri F. Laurette N. N. Annette D. John Q. Wolfgang M. François-Xavier E. (2008). Solubilization of inorganic phosphates and plant growth promotion by strains of *Pseudomonas fluorescens* isolated from acidic soils of Cameroon. Afr. J. Microbiol. Res. 2, 171–178. Available online at: https://academicjournals.org/journal/AJMR/article-full-text-pdf/233BC1A11313

[B105] Heydarian Z. Gruber M. Glick B. R. Hegedus D. D. (2018). Gene expression patterns in roots of camelina sativa with enhanced salinity tolerance arising from inoculation of soil with plant growth promoting bacteria producing 1-aminocyclopropane-1-carboxylate deaminase or expression the corresponding *acds* gene. Front. Microbiol. 9:1297 10.3389/fmicb.2018.01297 30013518 PMC6036250

[B106] Hiltner L. (1904). Über neuere Erfahrungen und Probleme auf dem Gebiete der Bodenbakteriologie unter besonderer Berücksichtigung der Gründüngung und Brache. Arbeit. Deutsch. Landwirtschaft. Gesellschaft. 98, 59–78.

[B107] Hossain K. Ismail N. (2015). Bioremediation and detoxification of pulp and paper mill effluent: a review. Res. J. Environ. Toxicol. 9, 113–134. 10.3923/rjet.2015.113.134

[B108] Huang C. P. Huang C. P. Morehart A. L. (1990). The removal of Cu (II) from dilute aqueous solutions by *Saccharomyces cerevisiae*. Water Res. 24, 433–439. 10.1016/0043-1354(90)90225-U

[B109] Huang H. Wu K. Khan A. Jiang Y. Ling Z. Liu P.. (2016). A novel *Pseudomonas gessardii* strain LZ-E simultaneously degrades naphthalene and reduces hexavalent chromium. Bioresour. Technol. 207, 370–378. 10.1016/j.biortech.2016.02.015 26901089

[B110] Hussain A. Arshad M. Zahir Z. A. Asghar M. (2015). Prospects of zinc solubilizing bacteria for enhancing growth of maize. Pak. J. Agri. Sci. 52, 915–922.

[B111] Hussain S. Hartley C. J. Shettigar M. Pandey G. (2016). Bacterial biodegradation of neonicotinoid pesticides in soil and water systems. FEMS Microbiol. Lett. 363:fnw252. 10.1093/femsle/fnw252 28003337

[B112] Hwang S. Ramirez N. Cutright T. J. Ju L. K. (2003). The role of soil properties in pyrene sorption and desorption. Water Air Soil Pollut. 143, 65–80. 10.1023/A:1022863015709

[B113] Iida M. Takemoto K. (2018). A. network biology-based approach to evaluating the effect of environmental contaminants on human interactome and diseases. Ecotoxicol. Environ. Saf. 160, 316–327. 10.1016/j.ecoenv.2018.05.065 29857236

[B114] Ijaz A. Imran A. Anwar ul Haq M. Khan Q. M. Afzal M. (2016). Phytoremediation: recent advances in plant-endophytic synergistic interactions. Plant Soil 405, 179–195. 10.1007/s11104-015-2606-2

[B115] Innerebner G. Knief C. Vorholt J. A. (2011). Protection of *Arabidopsis thaliana* against leaf-pathogenic *Pseudomonas syringae* by *Sphingomonas strainsina* controlled model system. Appl. Environ. Microbiol. 77, 3202–3210. 10.1128/AEM.00133-11 21421777 PMC3126462

[B116] Isaac P. Martínez F. L. Bourguignon N. Sánchez L. A. Ferrero M. A. (2015). Improved PAHs removal performance by a defined bacterial consortium of indigenous *Pseudomonas* and actinobacteria from Patagonia, Argentina. Int. Biodet. Biodeg. 101, 23–31. 10.1016/j.ibiod.2015.03.014

[B117] Jackson-Smith D. B. (2010). Toward Sustainable Agricultural Systems in the 21st Century. New York, NY: National Academies Press.

[B118] Jamil M. Ahamd M. Anwar F. Zahir Z. A. Kharal M. A. Nazli F. (2018). Inducing drought tolerance in wheat through combined use of L-tryptophan and *Pseudomonas fluorescens*. Pak. J. Agri. Sci. 55, 331–337. 10.21162/PAKJAS/18.4980

[B119] Jang H. N. Park S. B. Kim J. H. Seo Y. C. (2010). Combustion characteristics and emission of hazardous air pollutants in commercial fluidized bed combustors for sewage sludge, in Proceeding of the 13th International Conference on Fluidization–New Paradigm in Fluidization Engineering, Art. 77. New York, NY: ECI Digital Archives.

[B120] Jennings D. H. (1994). Translocation in fungal mycelia, in The Mycota: Growth, Differentiation and Sexuality, eds Wessels J. G. H. Meinhardt F. (Berlin: Springer), 163–173.

[B121] Jia Y. Yin H. Ye J. S. Peng H. He B. Y. Qin H. M.. (2008). Characteristics and pathway of Naphthalene degradation by *Pseudomonas* sp. N7. Huan Jing Ke Xue 29, 756–762. 18649540

[B122] Jin J. Yao J. Zhang Q. Liu J. (2016). Biodegradation of pyrene by pseudomonas sp. JPN_2_ and its initial degrading mechanism study by combining the catabolic nahAc gene and structure-based analyses. Chemosphere 164, 379–386. 10.1016/j.chemosphere.2016.08.113 27596825

[B123] Johnsen A. R. Wick L. Y. Harms H. (2005). Principles of microbial PAH-degradation in soil. Environ. Pollut. 133, 71–84. 10.1016/j.envpol.2004.04.015 15327858

[B124] Johnsen R. E. (1976). DDT metabolism in microbial systems. Residue Rev. 61, 1–28. 10.1007/978-1-4613-9401-3_1 778953

[B125] Joshi P. K. Yadav R. K. (2005). Effect of sewage on microbiological and chemical properties and crop growth in reclaimed alkali soil, in Proceedings of the International Conference on Soil, water and Environment Quality, Issues and Stratigies (New Delhi, India).

[B126] Kachhawa D. (2017). Microorganisms as a biopesticides. J. Entomol. Zool. Studies 5, 468–473.

[B127] Kadri T. Rouissi T. Brar S. K. Cledon M. Sarma S. Verma M. (2016). Biodegradation of polyaromatic hydrocarbons (PAHs) by fungal enzymes: a review. J. Environ. Sci. 51, 52–74. 10.1016/j.jes.2016.08.023 28115152

[B128] Kalyani D. C. Patil P. S. Jadhav J. P. Govindwar S. P. (2008). Biodegradation of reactive textile dye Red BLI by an isolated bacterium *Pseudomonas* sp. SUK1. Bioresour. Technol. 99, 4635–4641. 10.1016/j.biortech.2007.06.058 17765541

[B129] Kamali M. Khodaparast Z. (2015). Review on recent developments on pulp and paper mill wastewater treatment. Ecotoxicol. Environ. Saf. 114, 326–342. 10.1016/j.ecoenv.2014.05.005 24953005

[B130] Kamran S. Shahid I. Baig D. N. Rizwan M. Malik K. A. Mehnaz S. (2017). Contribution of zinc solubilizing bacteria in growth promotion and zinc content of wheat. Front. Microbiol. 8:2593. 10.3389/fmicb02593 29312265 PMC5743011

[B131] Kang B. G. Kim W. T. Yun H. S. Chang S. C. (2010). Use of plant growth-promoting rhizobacteria to control stress responses of plant roots. Plant Biotechnol. Rep. 4, 179–183. 10.1007/s11816-010-0136-1

[B132] Kannan K. Tanabe S. Williams R. J. Tatsukawa R. (1994). Persistent organochlorine residues in foodstuffs from Australia, Papua New Guinea and the Solomon Islands: contamination levels and human dietary exposure. Sci. Total Environ. 153, 29–49. 10.1016/0048-9697(94)90099-X 7939620

[B133] Karthik C. Oves M. Thangabalu R. Sharma R. Santhosh S. B. Arulselvi P. I. (2016). *Cellulosimicrobium funkei*-like enhances the growth of *Phaseolus vulgaris* by modulating oxidative damage under chromium(VI) toxicity. J. Adv. Res. 7, 839–850. 10.1016/j.jare.2016.08.007 27668092 PMC5026708

[B134] Khalid A. Akhtar M. J. Mahmood M. H. Arshad M. (2006). Effect of substrate-dependent microbial ethylene production on plant growth. Microbiology 75, 231–236. 10.1134/S0026261706020196 16758878

[B135] Khalid A. Arshad M. Crowley D. E. (2008a). Accelerated decolorization of structurally different azo dyes by newly isolated bacterial strains. Appl. Microbiol. Biotechnol. 78, 361–369. 10.1007/s00253-007-1302-4 18084755

[B136] Khalid A. Arshad M. Crowley D. E. (2008b). Decolorization of azo dyes by *Shewanella* sp. under saline conditions. Appl. Microbiol. Biotechnol. 79, 1053–1059. 10.1007/s00253-008-1498-y 18461315

[B137] Khalid A. Arshad M. Crowley D. E. (2009). Biodegradation potential of pure and mixed bacterial cultures for removal of 4-nitroaniline from textile dye wastewater. Water Res. 43, 1110–1116. 10.1016/j.watres.2008.11.045 19114284

[B138] Khan M. S. Ahmad E. Zaidi A. Oves M. (2013). Functional aspect of phosphate-solubilizing bacteria: importance in crop production, in Bacteria in Agrobiology: Crop Productivity, eds Maheshwari D. Saraf M. Aeron A. (Berlin; Heidelberg: Springer), 237–263.

[B139] Khan M. S. Zaidi A. Wani P. A. Ahemad M. Oves M. (2009a). Functional diversity among plant growth-promoting rhizobacteria: current status, in Microbial Strategies for Crop Improvement, eds Khan M. Zaidi A. Musarrat J. (Berlin; Heidelberg: Springer), 105–132.

[B140] Khan M. S. Zaidi A. Wani P. A. Oves M. (2009b). Role of plant growth promoting rhizobacteria in the remediation of metal contaminated soils. Environ. Chem. Lett. 7, 1–19. 10.1007/s10311-008-0155-0

[B141] Kharche V. K. Desai V. N. Pharande A. L. (2011). Effect of sewage irrigation on soil properties, essential nutrient and pollutant element status of soils and plants in a vegetable growing area around Ahmednagar city in Maharashtra. J. Indian Soc. Soil Sci. 59, 177–184.

[B142] Kim J. Rees D. C. (1994). Nitrogenase and biological nitrogen fixation. Biochemistry 33, 389–397. 10.1021/bi00168a001 8286368

[B143] Kim S. J. Kweon O. Jones R. C. Freeman J. P. Edmondson R. D. Cerniglia C. E. (2007). Degradation of 2,3-diethyl-5-methylpyrazine by a newly discovered bacterium, *Mycobacterium* sp. strain DM-11. J. Bacteriol. 189, 464–472. 10.1128/AEM.72.2.1437-1444.2006 17085566 PMC1797382

[B144] Kim Y. C. Jung H. Kim K. Y. Park S. K. (2008). An effective biocontrol bioformulation against *Phytophthora* blight of pepper using growth mixtures of combined chitinolytic bacteria under different field conditions. Eur. J. Plant Pathol. 120, 373–382. 10.1007/s10658-007-9227-4

[B145] Kirkeleit J. Riise T. Bråtveit M. Moen B. E. (2006). Benzene exposure on a crude oil production vessel. Ann. Occup. Hyg. 50, 123–129. 10.1093/annhyg/mei065 16371415

[B146] Kloepper J. W. Leong J. Teintze M. Schroth M. N. (1980). Enhanced plant growth by siderophores produced by plant growth-promoting rhizobacteria. Nature 286, 885–886. 10.1038/286885a0

[B147] Konieczny A. Kowalaska I. (2016). The role of arbuscular mycorrhiza in zinc uptake by lettuce grown at two phosphorus levels in the substrate. Agri. Food Sci. 25, 124–137. 10.23986/afsci.55534

[B148] Kuffner M. Puschenreiter M. Wieshammer G. Gorfer M. Sessitsch A. (2008). Rhizosphere bacteria affect growth and metal uptake of heavy metal accumulating willows. Plant Soil 304, 35–44. 10.1007/s11104-007-9517-9

[B149] Kulshrestha Y. Husain Q. (2007). Decolorization and degradation of acid dyes mediated by salt fractionated turnip (*Brassica rapa*) peroxidases. Toxicol. Environ Chem. 89, 255–267. 10.1080/02772240601081692

[B150] Kurade M. B. Waghmode T. R. Govindwar S. P. (2011). Preferential biodegradation of structurally dissimilar dyes from a mixture by *Brevibacillus laterosporus*. J. Hazard. Mater. 192, 1746–1755. 10.1016/j.jhazmat.2011.07.004 21803494

[B151] Lal R. Saxena D. M. (1982). Accumulation, metabolism, and effects of organochlorine insecticides on microorganisms. Microbiol. Rev. 46, 95–127. 6178010 10.1128/mr.46.1.95-127.1982 PMC373213

[B152] Langlois B. E. Collins J. A. Sides K. G. (1970). Some factors affecting degradation of organochlorine pesticides by bacteria. J. Dairy Sci. 53, 1671–1675. 10.3168/jds.S0022-0302(70)86461-X 4992932

[B153] Lapen D. R. Topp E. Edwards M. Sabourin L. Curnoe W. Gottschall N.. (2008). Effect of liquid municipal biosolid application method on tile and ground water quality. J. Environ. Qual. 37, 925–936. 10.2134/jeq2006.0486 18453415

[B154] Lichtfouse E. Navarrete M. Debaeke P. Souchere V. Alberola C. Menassieu J. (2009). Agronomy for sustainable agriculture. A review. Agron. Sustain. Dev. 29, 1–6. 10.1051/agro:2008054

[B155] Liu H. Wang X. Qi H. Wang Q. Chen Y. Li Q. (2017). The infection and impact of *Azorhizobium caulinodans* ORS571 on wheat (*Triticum aestivum* L.). PLoS ONE 12:e0187947. 10.1371/journal.pone.0187947 29190702 PMC5708735

[B156] Liu H. L. Chen B. Y. Lan Y. W. Cheng Y. C. (2004). Biosorption of Zn (II) and Cu (II) by the indigenous *Thiobacillus thiooxidans*. Chem. Eng. J. 97, 195–201. 10.1016/S1385-8947(03)00210-9

[B157] Lu L. Wu Q. (2017). Mycorrhizas promote plant growth, root morphology and chlorophyll production in white clover. Biotechnology 16, 34–39. 10.3923/biotech.2017.34.39

[B158] Lu L. Zhao M. Wang T. N. Zhao L. Y. Du M. H. Li T. L.. (2012). Characterization and dye decolorization ability of an alkaline resistant and organic solvents tolerant laccase from *Bacillus licheniformis* LS04. Bioresour. Technol. 115, 35–40. 10.1016/j.biortech.2011.07.111 21868217

[B159] Lugtenberg B. Kamilova F. (2009). Plant-growth-promoting rhizobacteria. Annu. Rev. Microbiol. 63, 541–556. 10.1146/annurev.micro.62.081307.162918 19575558

[B160] Ma Y. Rajkumar M. Vicente J. A. F. Freitas H. (2010). Inoculation of Ni-resistant plant growth promoting bacterium *Psychrobacter* sp. strain SRS8 for the improvement of nickel phytoextraction by energy crops. Int. J. Phytoremediation 13, 126–139. 10.1080/15226511003671403 21598781

[B161] Machuca A. Pereira G. Aguiar A. Milagres A. M. (2007). Metal-chelating compounds produced by ectomycorrhizal fungi collected from pine plantations. Lett. Appl. Microbiol. 44, 7–12. 10.1111/j.1472-765X.2006.02046.x 17209807

[B162] Maheshwari D. K. (2013). Bacteria in Agrobiology: Disease Management. Berlin: Springer.

[B163] Malghani S. Chatterjee N. Yu H. X. Luo Z. (2009). Isolation and identification of profenofos degrading bacteria. Braz. J. Microbiol. 40, 893–900. 10.1590/S1517-838220090004000021 24031438 PMC3768572

[B164] Malik A. (2004). Metal bioremediation through growing cells. Environ. Int. 30, 261–278. 10.1016/j.envint.2003.08.001 14749114

[B165] Marathe R. J. Phatake Y. B. Sonawane A. M. (2015). Bioprospecting of *Pseudomonas aeruginosa* for their potential to produce siderophore, process optimization and evaluation of its bioactivity. Int. J. Bioassays 4, 3667–3675. 10.21746/ijbio.2015.02.009

[B166] Matsumura F. Boush G. M. Tai A. (1968). Breakdown of dieldrin in the soil by a micro-organism. Nature 219, 965–967. 10.1038/219965a0 5673021

[B167] Mayak S. Tirosh T. Glick B. R. (2004). Plant growth-promoting bacteria confer resistance in tomato plants to salt stress. Plant Physiol. Biochem. 42, 565–572. 10.1016/j.plaphy.2004.05.009 15246071

[B168] Mazurier S. Corberand T. Lemanceau P. Raaijmakers J. M. (2009). Phenazine antibiotics produced by fuorescent pseudomonads contribute to natural soil suppressiveness to *Fusarium* wilt. ISME J. 3, 977–991. 10.1038/ismej.2009.33 19369971

[B169] McErlean C. Marchant R. Banat I. M. (2006). An evaluation of soil colonisation potential of selected fungi and their production of ligninolytic enzymes for use in soil bioremediation applications. Antonie van Leeuwenhoek 90, 147–158. 10.1007/s10482-006-9069-7 16820969

[B170] McKelvey W. Jacobson J. B. Kass D. Barr D. B. (2014). Biomonitoring of exposure to organophosphate pesticides. Environ. Health Perspect. 122, 1–2. 10.1289/ehp.1408444R 24984157 PMC4080521

[B171] Meena S. S. Sharma R. S. Gupta P. Karmakar S. Aggarwal K. K. (2016). Isolation and identification of *Bacillus megaterium* YB3 from an effluent contaminated site efficiently degrades pyrene. J. Basic Microbiol. 56, 369–378. 10.1002/jobm.201500533 26755240

[B172] Mineki S. Suzuki K. Iwata K. Nakajima D. Goto S. (2015). Degradation of polyaromatic hydrocarbons by fungi isolated from soil in Japan. Polycycl. Arom. Comp. 35, 120–128. 10.1080/10406638.2014.937007

[B173] Mishra V. Lal R. Srinivasan (2001). Enzymes and operons mediating xenobiotic degradation in bacteria. Crit. Rev. Microbiol. 27, 133–166. 10.1080/20014091096729 11450853

[B174] Mohamed M. S. (2009). Degradation of methomyl by the novel bacterial strain *Stenotrophomonas maltophilia* M1. Electron. J. Biotechnol. 12, 6–7. 10.2225/vol12-issue4-fulltext-11

[B175] Moharana P. C. Biswas D. R. Patra A. K. Datta S. C. Singh R. D. Bandyopadhyay K. K. (2014). Soil nutrient availability and enzyme activities under wheat-green gram crop rotation as affected by rock phosphate enriched compost and inorganic fertilizers. J. Indian Soc. Soil Sci. 62, 224–234.

[B176] Moreby S. J. Southway S. Barker A. Holland J. M. (2001). A comparison of the effect of new and established insecticides on nontarget invertebrates of winter wheat fields. Environ. Toxicol. Chem. 20, 2243–2254. 10.1002/etc.5620201017 11596757

[B177] Mullin C. A. Chen J. Fine J. D. Frazier M. T. Frazier J. L. (2015). The formulation makes the honey bee poison. Pestic. Biochem. Physiol. 120, 27–35. 10.1016/j.pestbp.2014.12.026 25987217

[B178] Mumtaz M. Z. Ahmad M. Jamil M. Asad S. A. Hafeez F. (2018). *Bacillus* strains as potential alternate for zinc biofortification of maize grains. Int. J. Agri. Biol. 20 10.17957/IJAB/15.0690

[B179] Mumtaz M. Z. Ahmad M. Jamil M. Hussain T. (2017). Zinc solubilizing *Bacillus* spp. potential candidates for biofortification in maize. Microbiol. Res. 202, 51–60. 10.1016/j.micres.2017.06.001 28647123

[B180] Nadeem S. M. Ahmad M. Zahir Z. A. Ashraf M. (2011). Microbial ACC-deaminase biotechnology: perspectives and applications in stress agriculture, in Bacteria in Agrobiology: Stress Management, ed Maheshwari D. K. (Berlin: Springer), 141–185.

[B181] Nadeem S. M. Ahmad M. Zahir Z. A. Javaid A. Ashraf M. (2014). The role of mycorrhizae and plant growth promoting rhizobacteria (PGPR) in improving crop productivity under stressful environments. Biotechnol. Adv. 32, 429–448. 10.1016/j.biotechadv.2013.12.005 24380797

[B182] Nadeem S. M. Ahmad M. Zahir Z. A. Kharal M. A. (2016). Role of phytohormones in stress tolerance of plants, in Plant, Soil and Microbes–Volume 2, Mechanisms and Molecular Interactions, eds Hakeem K. R. Akhtar M. S. (Cham: Springer), 385–421.

[B183] Nadeem S. M. Imran M. Naveed M. Khan M. Y. Ahmad M. Zahir Z. A.. (2017). Synergistic use of biochar, compost and plant growth-promoting rhizobacteria for enhancing cucumber growth under water deficit conditions. J. Sci. Food Agri. 97, 5139–5145. 10.1002/jsfa.8393 28436040

[B184] Nadeem S. M. Naveed M. Ahmad M. Zahir Z. A. (2015). Rhizosphere bacteria for crop production and improvement of stress tolerance: mechanisms of action, applications, and future prospects, in Plant Microbes Symbiosis: Applied Facets, ed Arora N. K. (New Delhi: Springer), 1–36.

[B185] Nadeem S. M. Zahir Z. A. Naveed M. Arshad M. (2009). Rhizobacteria containing ACC-deaminase confer salt tolerance in maize grown on salt-affected fields. J. Microbiol. 55, 1302–1309. 10.1139/w09-092 19940939

[B186] Narwal R. P. Singh M. Gupta A. P. (1988). Effect of different sources of irrigation on the physico-chemical properties of soil. Indian J. Environ. Agri. 3, 27–34.

[B187] Neilands J. B. (1995). Siderophores: structure and function of microbial iron transport compounds. J. Biol. Chem. 270, 26723–26726. 10.1074/jbc.270.45.26723 7592901

[B188] Nies D. H. (1999). Microbial heavy metal resistance. Appl. Microbiol. Biotechnol. 51, 730–750. 10.1007/s002530051457 10422221

[B189] Nwinyi O. C. Ajayi O. O. Amund O. O. (2016). Degradation of polynuclear aromatic hydrocarbons by two strains of *Pseudomonas*. Brazilian J. Microbiol. 47, 551–562. 10.1016/j.bjm.2016.04.026 27245129 PMC4927684

[B190] Ojuederie O. B. Babalola O. O. (2017). Microbial and plant-assisted bioremediation of heavy metal polluted environments: a review. Int. J. Environ. Res. Public Health 14:1504. 10.3390/ijerph14121504 29207531 PMC5750922

[B191] Olguín E. J. (2003). Phycoremediation: key issues for cost-effective nutrient removal processes. Biotechnol. Adv. 22, 81–91. 10.1016/S0734-9750(03)00130-7 14623045

[B192] Ortega-Gonzalez D. K. Martinez-Gonzalez G. Flores C. M. Zaragoza D. Cancino-Diaz J. C. Cruz-Maya J. A. (2015). *Amycolatopsis* sp. Poz14 isolated from oil-contaminated soil degrades polycyclic aromatic hydrocarbons. Int. Biodet. Biodeg. 99, 650–173. 10.1016/j.ibiod.2015.01.008

[B193] Osborn R. K. Haydock P. P. J. Edwards S. G. (2010). Isolation and identification of oxamyl-degrading bacteria from UK agricultural soils. Soil Biol. Biochem. 42, 998–1000. 10.1016/j.soilbio.2010.01.016

[B194] O'Sullivan D. J. O'Gara F. (1992). Traits of fluorescent *Pseudomonas* spp. involved in suppression of plant root pathogens. Microbiol. Rev. 56, 662–676. 1480114 10.1128/mr.56.4.662-676.1992 PMC372893

[B195] Oves M. Khan M. S. Zaidi A. (2013). Chromium reducing and plant growth promoting novel strain *Pseudomonas aeruginosa* OSG41 enhance chickpea growth in chromium amended soils. Eur. J. Soil Biol. 56, 72–83. 10.1016/j.ejsobi.2013.02.002

[B196] Oves M. Khan M. S. Zaidi A. Ahmad E. (2012). Soil contamination, nutritive value, and human health risk assessment of heavy metals: an overview, in Toxicity of Heavy Metals to Legumes and Bioremediation, eds Zaidi A. Wani P. Khan M. (Vienna: Springer), 1–27.

[B197] Ozdal M. Ozdal O. G. Algur O. F. (2016). Isolation and characterization of α-endosulfan degrading bacteria from the microflora of cockroaches. Pol. J. Microbiol. 65, 63–68. 10.5604/17331331.1197325 27281995

[B198] Pal A. Paul A. K. (2004). Aerobic chromate reduction by chromium-resistant bacteria isolated from serpentine soil. Microbiol. Res. 159, 347–354. 10.1016/j.micres.2004.08.001 15646381

[B199] Pandey A. Singh P. Iyengar L. (2007). Bacterial decolorization and degradation of azo dyes. Int. Biodet. Biodeg. 59, 73–84. 10.1016/j.ibiod.2006.08.006

[B200] Pandey R. K. Tewari S. Tewari L. (2018). Lignolytic mushroom Lenzites elegans WDP2: Laccase production, characterization, and bioremediation of synthetic dyes. Ecotoxicol. Environ. Saf. 158, 50–58. 10.1016/j.ecoenv.2018.04.003 29656164

[B201] Parte S. G. Mohekar A. D. Kharat A. S. (2017). Microbial degradation of pesticide: a review. Afr. J. Microbiol. Res. 11, 992–1012. 10.5897/AJMR2016.8402

[B202] Patten C. L. Glick B. R. (2002). Role of *Pseudomonas putida* indole acetic acid in development of the host plant root system. Appl. Environ. Microbiol. 68, 3795–3801. 10.1128/AEM.68.8.3795-3801.2002 12147474 PMC124051

[B203] Pérez E. Sulbarán M. Ball M. M. Yarzabál L. A. (2007). Isolation and characterization of mineral phosphate-solubilizing bacteria naturally colonizing a limonitic crust in the southeastern Venezuelan region. Soil Biol. Biochem. 39, 2905–2914. 10.1016/j.soilbio.2007.06.017

[B204] Perotti E. B. R. Pidello A. (2012). Plant-soil-microorganism interactions on nitrogen cycle: *Azospirillum* inoculation, in Advances in Selected Plant Physiology Aspects, ed Montanaro G. (Shanghai: Intech), 189–208.

[B205] Picazevicz A. A. C. Kusdra J. F. Moreno A. D. L. (2017). Maize growth in response to *Azospirillum brasilense, Rhizobium tropici*, molybdenum and nitrogen. Rev. Bras. Eng. Agric. Ambient 21, 623–627. 10.1590/1807-1929/agriambi.v21n9p623-627

[B206] Pieterse C. M. Leon-Reyes A. van der Ent S. van Wees S. C. (2009). Networking by small-molecule hormones in plant immunity. Nat. Chem. Biol. 5, 308–316. 10.1038/nchembio.164 19377457

[B207] Poeton T. S. Stensel H. D. Strand S. E. (1999). Biodegradation of polyaromatic hydrocarbons by marine bacteria: effect of solid phase on degradation kinetics. Water Res. 33, 868–880. 10.1016/S0043-1354(98)00232-2

[B208] Prabhukarthikeyan R. Saravanakumar D. Raguchander T. (2014). Combination of endophytic *Bacillus* and *Beauveria* for the management of *Fusarium* wilt and fruit borer in tomato. Pest Manage. Sci. 70, 1742–50. 10.1002/ps.3719 24376014

[B209] Prasad A. Rao K. V. B. (2011). Physicochemical analysis of textile effluent and decolorization of textile azo dye by *Bacillus endophyticus* strain VITABR13. IIOAB J. 2, 55–62.

[B210] Qiu X. Wu P. Zhang H. Li M. Yan Z. (2009). Isolation and characterization of *Arthrobacter* sp. HY2 capable of degrading a high concentration of *p*-nitrophenol. Bioresour. Technol. 100, 5243–5248. 10.1016/j.biortech.2009.05.056 19540107

[B211] Qu C. Du H. Ma M. Chen W. Cai P. Huang Q. (2018). Pb Sorption on montmorillonite-bacteria composites: a combination study by XAFS, ITC and SCM. Chemosphere 200, 427–436. 10.1016/j.chemosphere.2018.02.136 29501033

[B212] Qu C. Ma M. Chen W. Cai P. Huang Q. (2017b). Surface complexation modeling of Cu(II) sorption to montmorillonite-bacteria composites. Sci. Total Environ. 607–608, 1408–1418. 10.1016/j.scitotenv.2017.07.068 28738531

[B213] Qu C. Ma M. Chen W. Cai P. Yu X. Feng. (2017a). Modeling of Cd adsorption to goethite-bacteria composites. Chemosphere 193, 943–950. 10.1016/j.chemosphere.2017.11.100 29874770

[B214] Raaijmakers J. M. Vlami M. de Souza J. T. (2002). Antibiotic production by bacterial biocontrol agents. Antonie van Leeuwenhoek 81, 537–547. 10.1023/A:1020501420831 12448749

[B215] Raja D. Takankhar V. J. (2018). Response of liquid biofertilizers (*Bradyrhizobium* and PSB) on nutrient content in soybean. IJCMAS 7, 3701–3706. 10.20546/ijcmas.2018.705.428

[B216] Rajendhran J. Gunasekaran P. (2008). Strategies for accessing soil metagenome for desired applications. Biotechnol. Adv. 26, 576–590. 10.1016/j.biotechadv.2008.08.002 18786627

[B217] Rajkumar M. Ae N. Prasad M. N. V. Freitas H. (2010). Potential of siderophore-producing bacteria for improving heavy metal phytoextraction. Trends Biotechnol. 28, 142–149. 10.1016/j.tibtech.2009.12.002 20044160

[B218] Ramesh A. Sharma S. K. Sharma M. P. Yadav N. Joshi O. P. (2014). Inoculation of zinc solubilizing *Bacillus aryabhattai* strains for improved growth, mobilization and biofortification of zinc in soybean and wheat cultivated in vertisols of central India. Appl. Soil Ecol. 73, 87–96. 10.1016/j.apsoil.2013.08.009

[B219] Rasche F. Cadisch G. (2013). The molecular microbial perspective of organic matter turnover and nutrient cycling in tropical agroecosystems–What do we know? Biol. Fertil. Soils 49, 251–262. 10.1007/s00374-013-0775-9

[B220] Rashid M. I. Mujawar L. H. Shahzad T. Almeelbi T. Ismail I. M. I. Oves M. (2016). Bacteria and fungi can contribute to nutrients bioavailability and aggregate formation in degraded soils. Microbiol. Res. 183, 26–41. 10.1016/j.micres.2015.11.007 26805616

[B221] Rayu S. Nielsen U. N. Nazaries L. Singh B. K. (2017). Isolation and molecular characterization of novel chlorpyrifos and 3,5,6-trichloro-2-pyridinol-degrading bacteria from sugarcane farm soils. Front. Microbiol. 8:518. 10.3389/fmicb.2017.00518 28421040 PMC5378769

[B222] Reddy G. V. S. Rafi M. M. Kumar S. R. Khayalethu N. Rao D. M. Manjunatha B.. (2016). Optimization study of 2-hydroxyquinoxaline (2-HQ) biodegradation by *Ochrobactrum* sp. HQ1. Biotechnology 6, 51–61. 10.1007/s13205-015-0358-6 28330121 PMC4746200

[B223] Rochkind-Dubinsky M. L. Sayler G. S. Blackburn J. W. (1986). Microbiological Decomposition of Chlorinated Aromatic Compounds, Vol. 18, Microbiology Series, Chapter 18. New York, NY: Marcel Dekker Inc.

[B224] Rodrigues A. A. Forzani M. V. (2016). Isolation and selection of plant growth-promoting bacteria associated with sugarcane. Pesq. Agropec. Trop. Goiania 46, 149–158. 10.1590/1983-40632016v4639526

[B225] Rodriguez H. Vessely S. Shah S. Glick B. R. (2008). Effect of a nickel-tolerant ACC deaminase-producing *Pseudomonas* strain on growth of non-transformed and transgenic canola plants. Curr. Microbiol. 57, 170–174. 10.1007/s00284-008-9181-1 18560939

[B226] Rosenblueth M. Martínez-Romero E. (2006). Bacterial endophytes and their interactions with hosts. Mol. Plant Microbe Interact. 19, 827–37. 10.1094/MPMI-19-0827 16903349

[B227] Rousidou K. Chanika E. Georgiadou D. Soueref E. Katsarou D. Kolovos P.. (2016). Isolation of oxamyl-degrading bacteria and identification of *cehA* as a novel oxamyl hydrolase gene. Front. Microbiol. 7:616. 10.3389/fmicb.2016.00616 27199945 PMC4850150

[B228] Rubio L. M. Ludden P. W. (2008). Biosynthesis of the iron-molybdenum cofactor of nitrogenase. Annu. Rev. Microbiol. 62, 93–111. 10.1146/annurev.micro.62.081307.162737 18429691

[B229] Saleem M. Arshad M. Hussain S. Bhatti A. S. (2007). Perspective of plant growth promoting rhizobacteria (PGPR) containing ACC deaminase in stress agriculture. J. Indian Microbiol. Biotechnol. 34, 635–648. 10.1007/s10295-007-0240-6 17665234

[B230] Salloum M. S. Menduni M. F. Luna C. M. (2017). A differential capacity of arbuscular mycorrhizal fungal colonization under well-watered conditions and its relationship with drought stress mitigation in unimproved vs. improved soybean genotypes. Botany 96, 135–44. 10.1139/cjb-2017-0137

[B231] Saptanmasi B. Karayilanoglu T. Kenar L. Serdar M. Kose S. Aydin A. (2008). Bacterial biodegradation of aldicarb and determination of bacterium which has the most biodegradative effect. Turk. J. Biochem. 33, 209–214.

[B232] Saravanan V. S. Madhaiyan M. Thangaraju M. (2007). Solubilization of zinc compounds by the diazotrophic, plant growth promoting bacterium *Gluconacetobacter diazotrophicus*. Chemosphere 66, 1794–1798. 10.1016/j.chemosphere.2006.07.067 16956644

[B233] Saravanan V. S. Subramoniam S. R. Raj S. A. (2003). Assessing *in vitro* solubilization of different zinc solubilizing bacterial (ZBS) strains. Braz. J. Microbiol. 35, 121–125. 10.1590/S1517-83822004000100020

[B234] Saroj S. Kumar K. Pareek N. Prasad R. Singh R. P. (2014). Biodegradation of azo dyes Acid Red 183, Direct Blue 15 and Direct Red 75 by the isolate *Penicillium oxalicum* SAR-3. Chemosphere 107, 240–248. 10.1016/j.chemosphere.2013.12.049 24418068

[B235] Schmidt W. (1999). Mechanisms and regulation of reduction-based iron uptake in plants. New Phytol. 141, 1–26. 10.1046/j.1469-8137.1999.00331.x

[B236] Senthilkumar S. Perumalsamy M. Prabhu H. J. (2014). Decolourization potential of white-rot fungus *Phanerochaete chrysosporium* on synthetic dye bath effluent containing amido black 10B. J. Saudi Chem. Soc. 18, 845–853. 10.1016/j.jscs.2011.10.010

[B237] Seo J. S. Keum Y. S. Hu Y. Lee S. E. Li Q. X. (2006). Phenanthrene degradation in *Arthrobacter* sp. P1-1: Initial 1,2-,3,4- and 9,10-dioxygenation, and meta- and ortho-cleavages of naphthalene-1,2-diol after its formation from naphthalene-1,2–dicarboxylic acid and hydroxyl naphthoic acids. Chemosphere 65, 2388–2394. 10.1016/j.chemosphere.2006.04.067 16777186

[B238] Sessitsch A. Reiter B. Berg G. (2004). Endophytic bacterial communities of field-grown potato plants and their plant-growth-promoting and antagonistic abilities. Can. J. Microbiol. 50, 239–249. 10.1139/w03-118 15213748

[B239] Shahsavar A. R. Refahi A. Zarei M. Aslmoshtagh E. (2016). Analysis of the effects of *Glomus etunicatum* fungi and *Pseudomonas fluorescence* bacteria symbiosis on some morphological and physiological characteristics of Mexican lime (*Citrus aurantifolia* L.) under drought stress conditions. Adv. Hort. Sci. 30, 39–45. 10.13128/ahs-18700

[B240] Shaikh S. Saraf M. (2017). Biofortification of *Triticum aestivum* through the inoculation of zinc solubilizing plant growth promoting rhizobacteria in field experiment. Biocatal. Agric. Biotechnol. 9, 120–126. 10.1016/j.bcab.2016.12.008

[B241] Shao Y. Wang Y. Wu X. Xu X. Kong S. Tong L. (2015). Biodegradation of PAHs by *Acinetobacter* isolated from karst groundwater in a coal-mining area. Environ. Earth Sci. 73, 7479–7488. 10.1007/s12665-014-3920-3

[B242] Sharma A. Johri B. N. Sharma A. K. Glick B. R. (2003). Plant growth-promoting bacterium *Pseudomonas* sp. strain GRP3 influences iron acquisition in mung bean (*Vigna radiata* L. Wilzeck). Soil Biol. Biochem. 35, 887–894. 10.1016/S0038-0717(03)00119-6

[B243] Sharma R. Chandra S. Singh A. Singh K. (2014). Degradation of pulp and paper mill effluents. IIOAB J. 5, 6–12.

[B244] Shelton J. F. Picciotto I. H. Pessah I. N. (2012). Tipping the balance of autism risk: potential mechanisms linking pesticides and autism. Environ. Health Perspect. 120, 944–951. 10.1289/ehp.1104553 22534084 PMC3404662

[B245] Sheng X. F. Xia J. J. Jiang C. Y. He L. Y. Qian M. (2008). Characterization of heavy metal-resistant endophytic bacteria from rape (*Brassica napus*) roots and their potential in promoting the growth and lead accumulation of rape. Environ. Pollut. 156, 1164–70. 10.1016/j.envpol.2008.04.007 18490091

[B246] Shi S. M. Chen K. Gao Y. Liu B. Yang X. H. Huang X. Z.. (2016). Arbuscular mycorrhizal fungus species dependency governs better plant physiological characteristics and leaf quality of mulberry (*Morus alba* L.) seedlings. Front. Microbiol. 7:1030. 10.3389/fmicb.2016.01030 27446063 PMC4923160

[B247] Sihag S. Pathak H. Jaroli D. P. (2014). Factors affecting the rate of biodegradation of polyaromatic hydrocarbons. Int. J. Pure App. Biosci. 2, 185–202. Availbale online at: http://www.ijpab.com/form/2014%20Volume%202,%20issue%203/IJPAB-2014-2-3-185-202.pdf

[B248] Singh D. Geat N. Rajawat M. V. S. Prasanna R. Saxena A. K. Kaushik R. (2017). Isolation and characterization of plant growth promoting endophytic diazotrophic bacteria from wheat genotypes and their influence on plant growth promotion. Int. J. Curr. Microbiol. Appl. Sci. 6, 1533–1540. 10.20546/ijcmas.2017.604.188

[B249] Singh D. K. (2008). Biodegradation and bioremediation of pesticide in soil: concept, method and recent developments. Indian J. Microbiol. 48, 35–40. 10.1007/s12088-008-0004-7 23100698 PMC3450205

[B250] Spina F. Cecchi G. Landinez-Torres A. Pecoraro L. Russo F. Wu B. (2018). Fungi as a toolbox for sustainable bioremediation of pesticides in soil and water. Plant Biosyst. 152, 474–488. 10.1080/11263504.2018.1445130

[B251] Srivastava S. Ahmad A. H. Thakur I. S. (2007). Short communication: removal of chromium and pentachlorophenol from tannery effluents. Bioresour. Technol. 98, 1128–1132. 10.1016/j.biortech.2006.04.011 16762546

[B252] Steenhoudt O. Vanderleyden J. (2000). *Azospirillum*, a free-living nitrogen fixing bacterium closely associated with grasses: genetic, biochemical and ecological aspects. FEMS Microbiol. Rev. 24, 487–506. 10.1111/j.1574-6976.2000.tb00552.x 10978548

[B253] Stephen J. Jisha M. S. (2009). Buffering reduces phosphate solubilizing ability of selected strains of bacteria. World J. Agric. Sci. 5, 135–137.

[B254] Sturz A. V. Christie B. R. Nowak J. (2000). Bacterial endophytes: potential role in developing sustainable systems of crop production. Crit. Rev. Plant Sci. 19, 1–30. 10.1080/07352680091139169

[B255] Sumathi S. Phatak V. (1999). Fungal treatment of bagasse based pulp and paper mill wastes. Environ. Technol. 20, 93–98. 10.1080/09593332008616797

[B256] Surekha R. M. Lakshmi P. K. L. Suvarnalatha D. Jaya M. Aruna S. Jyothi K. (2008). Isolation and characterization of a chlorpyrifos degrading bacterium from agricultural soil and its growth response. Afr. J. Microbiol. Res. 2, 26–31.

[B257] Taiz L. Zeiger E. (2000). Plant Physiology, 2nd Edn. San Francisco, CA: Benjamin Cumings Publishing Company.

[B258] Telke A. A. Ghodake G. S. Kalyani D. C. Dhanve R. S. Govindwar S. P. (2011). Biochemical characteristics of a textile dye degrading extracellular laccase from a *Bacillus* sp. ADR Bioresour. Technol. 102, 1752–1756. 10.1016/j.biortech.2010.08.086 20855194

[B259] Teng Y. Luo Y. Sun M. Liu Z. Li Z. Christie P. (2010). Effect of bioaugmentation by *Paracoccus* sp. strain HPD-2 on the soil microbial community and removal of polycyclic aromatic hydrocarbons from an aged contaminated soil. Bioresour. Technol. 101, 3437–3443. 10.1016/j.biortech.2009.12.088 20093016

[B260] Tiku D. K. Kumar A. Chaturvedi R. Makhijani S. D. Manoharan A. Kumar R. (2010). Holistic bioremediation of pulp mill effluents using autochthonous bacteria. Int. Biodet. Biodeg. 64, 173–183. 10.1016/j.ibiod.2010.01.001

[B261] Timmusk S. Behers L. Muthoni J. Muraya A. Aronsson A. C. (2017). Perspectives and challenges of microbial application for crop improvement. Front. Plant Sci. 8:49. 10.3389/fpls.2017.00049 28232839 PMC5299024

[B262] Torre-Ruiz N. D. L. Ruiz-Valdiviezo V. M. Rincón-Molina C. I. Rodríguez-Mendiola M. Arias-Castroa C. Gutiérrez-Miceli F. A. (2016). Effect of plant growth-promoting bacteria on the growth and fructan production of *Agave americana* L. Braz. J. Microbiol. 47, 587–596. 10.1016/j.bjm.2016.04.010 27268113 PMC4927679

[B263] Uqab B. Mudasir S. Nazir R. (2016). Review on bioremediation of pesticides. J. Biorem. Biodegrad. 7, 1–5. 10.4172/2155-6199.1000343

[B264] Uyttebroek M. Vermeir S. Wattiau P. Ryngaert A. Springael D. (2007). Characterization of cultures enriched from acidic polycyclic aromatic hydrocarbon-contaminated soil for growth on pyrene at low pH. Appl. Environ. Microbiol. 73, 3159–3164. 10.1128/AEM.02837-06 17369339 PMC1907120

[B265] Vansuyt G. Robin A. Briat J. F. Curie C. Lemanceau P. (2007). Iron acquisition from Fe-pyoverdine by *Arabidopsis thaliana*. Mol. Plant Microbe Interact. 20, 441–447. 10.1094/MPMI-20-4-0441 17427814

[B266] Verhagen B. W. Glazebrook J. Zhu T. Chang H. S. van Loon L. C. Pieterse C. M. J. (2004). The transcriptome of rhizobacteria-induced systemic resistance in *Arabidopsis*. Mol. Plant Microbe Interact. 17, 895–908. 10.1094/MPMI.2004.17.8.895 15305611

[B267] Vidali M. (2001). Bioremediation. An overview. Pure Appl. Chem. 73, 1163–1172. 10.1351/pac200173071163

[B268] Viscardi S. Ventorino V. Duran P. Maggio A. De Pascale S. Mora M. L. (2016). Assessment of plant growth promoting activities and abiotic stress tolerance of *Azotobacter chroococcum* strains for a potential use in sustainable agriculture. J. Soil Sci. Plant Nutr. 16, 848–863. 10.4067/S0718-95162016005000060

[B269] Vives-Peris V. Gomez-Cadenas A. Perez-Clemente R. M. (2018). Salt stress alleviation in citrus plants by plant growth-promoting rhizobacteria *Pseudomonas putida* and *Novosphingobium* sp. Plant Cell Rep. 30, 1–3. 10.1007/s00299-018-2328-z 30062625

[B270] Wahid A. Nasir M. G. A. Ahmad S. S. (2000). Effects of water pollution on growth and yield of soybean. Acta Sci. 10, 51–58. 10.1016/0378-4290(93)90062-R

[B271] Wakatsuki T. (1995). Metal oxidoreduction by microbial cells. J. Industrial Microbiol. Biotechnol. 14, 169–177. 10.1007/BF01569900 7766210

[B272] Wang C. Sun H. Li J. Li Y. Zhang Q. (2009). Enzyme activities during degradation of polycyclic aromatic hydrocarbons by white rot fungus *Phanerochaete chrysosporium* in soils. Chemosphere 77, 733–738. 10.1016/j.chemosphere.2009.08.028 19751947

[B273] Wang N. Du H. Huang Q. Cai P. Rong X. Feng X. (2016). Surface complexation modeling of Cd(II) sorption to montmorillonite, bacteria, and their composite. Biogeosciences 13, 5557–5566. 10.5194/bg-13-5557-2016

[B274] Wang W. Wang L. Shao Z. (2018b). Polycyclic aromatic hydrocarbon degradation pathways of the obligate marine PAH-degrader, *Cycloclasticus* sp. P1. Appl. Environ. Microbiol. 84:e01261–18. 10.1128/AEM.01261-18 30171002 PMC6193391

[B275] Wang Y. Brown H. N. Crowley D. E. Szaniszlo P. J. (1993). Evidence for direct utilization of a siderophore, ferrioxamine B, in axenically grown cucumber. Plant Cell Environ. 16, 579–585. 10.1111/j.1365-3040.1993.tb00906.x

[B276] Wang Y. Li H. Feng G. Du L. Zeng D. (2017). Biodegradation of diuron by an endophytic fungus Neurospora intermedia DP8-1 isolated from sugarcane and its potential for remediating diuron-contaminated soils. PLoS ONE 12:e0182556. 10.1371/journal.pone.0182556 28809955 PMC5557362

[B277] Wang Y. Wang M. Li Y. Wu A. Huang J. (2018a). Effects of arbuscular mycorrhizal fungi on growth and nitrogen uptake of *Chrysanthemum morifolium* under salt stress. PLoS ONE 13:e0196408. 10.1371/journal.pone.0196408 29698448 PMC5919403

[B278] Wani P. A. Khan M. S. (2010). *Bacillus* species enhance growth parameters of chickpea (*Cicer arietinum* L.) in chromium stressed soils. Food Chem. Toxicol. 48, 3262–3267. 10.1016/j.fct.2010.08.035 20813149

[B279] Watson S. L. (2014). Assessing the Impacts of Unrestricted Pesticide Use in Small-Scale Agriculture on Water Quality and Associated Human Health and Ecological Implications in an Indigenous Village in RURAL PANAMÁ. Master's thesis. Tampa, FL: University of South Florida.

[B280] Wienkoop S. Sistani N. R. Kaul H. P. Desalegn G. (2017). *Rhizobium* impacts on seed productivity, quality, and protection of *Pisum sativum* upon disease stress caused by *Didymella pinodes*: phenotypic, proteomic, and metabolomic traits. Front. Plant Sci. 8:1961. 10.3389/fpls.2017.01961 29204150 PMC5699443

[B281] Wilmowicz E. Kesy J. Kopcewicz J. (2008). Ethylene and ABA interactions in the regulation of flower induction in *Pharbitis nil*. J. Plant Physiol. 165, 1917–1928. 10.1016/j.jplph.2008.04.009 18565620

[B282] Wu Y. Li T. Yang L. (2012). Mechanisms of removing pollutants from aqueous solutions by microorganisms and their aggregates: a review. Bioresour. Technol. 107, 10–18. 10.1016/j.biortech.2011.12.088 22257855

[B283] Xia L. Xu X. Zhu W. Huang Q. Chen W. (2015). A comparative study on the biosorption of Cd^2+^ onto *Paecilomyces lilacinus* XLA and *Mucoromycote* sp. XLC. Int. J. Mol. Sci. 16, 15670–15687. 10.3390/ijms160715670 26184169 PMC4519919

[B284] Xia X. H. Yu H. Yang Z. F. Huang G. H. (2006). Biodegradation of polycyclic aromatic hydrocarbons in the natural waters of the Yellow River: Effects of high sediment content on biodegradation. Chemosphere 65, 457–466. 10.1016/j.chemosphere.2006.01.075 16540147

[B285] Xiao P. Mori T. Kamei I. Kondo R. (2010). Metabolism of organochlorine pesticide heptachlor and its metabolite heptachlor epoxide by white-rot fungi, belonging to genus *Phlebia*. FEMS Microbiol. Lett. 314, 140–146. 10.1111/j.1574-6968.2010.02152.x 21087297

[B286] Xiao Y. Wang X. Chen W. Huang Q. (2017). Isolation and identification of three potassium-solubilizing bacteria from rape rhizospheric soil and their effects on ryegrass. Geomicrobiol. J. 34, 873–880. 10.1080/01490451.2017.1286416

[B287] Xu J.-L. Wu J. Wang Z.-C. Wang K. Li M.-Y. Jiang J.-D. (2009). Isolation and characterization of a methomyl-degrading *Paracoccus* sp. Mdw-1. Pedosphere 19, 238–243. 10.1016/S1002-0160(09)60113-2

[B288] Xu X. Xia L. Chen W. Huang Q. (2017). Detoxification of hexavalent chromate by growing *Paecilomyces lilacinus* XLA. Environ. Poll. 225, 47–54. 10.1016/j.envpol.2017.03.039 28347903

[B289] Xu X. Xia L. Zhu W. Zhang Z. Huang Q. Chen W. (2015). Role of *Penicillium chrysogenum* XJ-1 in the detoxification and bioremediation of cadmium. Front. Microbiol. 6:1422. 10.3389/fmicb.2015.01422 26733967 PMC4685053

[B290] Xu Y. Zhou N. (2017). Microbial remediation of aromatics-contaminated soil. Front. Environ. Sci. Eng. 11:894 10.1007/s11783-017-0894-x

[B291] Yan Q.-X. Hong Q. Han P. Dong X.-J. Shen Y.-J. Li S.-P. (2007). Isolation and characterization of a carbofuran-degrading strain *Novosphingobium* sp. FND-3. FEMS Microbiol. Lett. 271, 207–213. 10.1111/j.1574-6968.2007.00718.x 17425661

[B292] Yilmaz E. I. (2003). Metal tolerance and biosorption capacity of *Bacillus circulans* strain EB1. Microbiology 154, 409–415. 10.1016/S0923-2508(03)00116-5 12892847

[B293] Yu C. L. Summers R. M. Li Y. Mohanty S. K. Subramanian M. Rope R. M. (2015). Rapid identification and quantitative validation of a caffeine-degrading pathway in *Pseudomonas* sp. CES. J. Proteome Res. 14, 95–106. 10.1021/pr500751w 25350919

[B294] Yuan S. Y. Chang J. S. (2001). Biodegradation of phenanthrene in river sediment. Chemosphere 43, 273–278. 10.1016/S0045-6535(00)00139-9 11302571

[B295] Yuanfan H. Jin Z. Qing H. Qian W. Jiandong J. Shunpeng L. (2010). Characterization of a fenpropathrin-degrading strain and construction of a genetically engineered microorganism for simultaneous degradation of methyl parathion and fenpropathrin. J. Environ. Manage. 91, 2295–2300. 10.1016/j.jenvman.2010.06.010 20624669

[B296] Zahir Z. A. Munir A. Asghar H. N. Shaharoona B. Arshad M. (2008). Effectiveness of rhizobacteria containing ACC-deaminase for growth promotion of pea (*Pisum sativum*) under drought conditions. J. Microbiol. Biotechnol. 18, 958–963. 18633298

[B297] Zahir Z. A. Naveed M. Zafar M. I. Rehman H. S. Arshad M. Khalid M. (2007). Evaluation of composted organic waste enriched with nitrogen and L-Tryptophan for improving growth and yield of wheat (*Triticum aestivum* L.). Pak. J. Bot. 39, 1739–1749.

[B298] Zahir Z. A. Shah M. K. Naveed M. Akhter M. J. (2010). Substrate-dependent auxin production by *Rhizobium phaseoli* improves the growth and yield of *Vigna radiata* L. under salt stress conditions. J. Microbiol. Biotechnol. 20, 1288–1294. 10.4014/jmb.1002.02010 20890093

[B299] Zahran H. H. (2001). Rhizobia from wild legumes: diversity, taxonomy, ecology, nitrogen fixation and biotechnology. J. Biotechnol. 91, 143–153. 10.1016/S0168-1656(01)00342-X 11566386

[B300] Zaidi A. Khan M. S. Ahemad M. Oves M. Wani P. A. (2009). Recent advances in plant growth promotion by phosphate-solubilizing microbes, in Microbial Strategies for Crop Improvement, eds Khan M. Zaidi A. Musarrat J. (Berlin; Heidelberg: Springer), 23–50.

[B301] Zhang F. Yediler A. Liang X. Kettrup A. (2004). Effects of dye additives on the ozonation process and oxidation by-products: a comparative study using hydrolyzed CI Reactive Red 120. Dyes Pigm. 60, 1–7. 10.1016/S0143-7208(03)00111-6

[B302] Zhang H. S. Wu X. H. Li G. Qin P. (2011a). Interactions between arbuscular mycorrhizal fungi and phosphate-solubilizing fungus (*Mortierella* sp.) and their effects on *Kostelelzkya virginica* growth and soil enzyme activities of rhizosphere and bulk soils at different salinities. Biol. Fertil. Soils 47, 543–554. 10.1007/s00374-011-0563-3

[B303] Zhang J. Jia W. Yang J. Ismail A. M. (2006). Role of ABA in integrating plant responses to drought and salt stresses. Field Crop. Res. 97, 111–119. 10.1016/j.fcr.2005.08.018

[B304] Zhang R. Xu X. Chen W. Huang Q. (2016). Genetically engineered *Pseudomonas putida* X3 strain and its potential ability to bioremediate soil microcosms contaminated with methyl parathion and cadmium. Appl. Microbiol. Biotechnol. 100, 1987–1997. 10.1007/s00253-015-7099-7 26521245

[B305] Zhang Y. F. He L. Y. Chen Z. J. Wang Q. Y. Qian M. Sheng X. F. (2011b). Characterization of ACC deaminase-producing endophytic bacteria isolated from copper-tolerant plants and their potential in promoting the growth and copper accumulation of *Brassica napus*. Chemosphere 83, 57–62. 10.1016/j.chemosphere.2011.01.041 21315404

[B306] Zhou H. Wang H. Huang Y. Fang T. (2016). Characterization of pyrene degradation by halophilic *Thalassospira* sp. strain TSL5-1 isolated from the coastal soil of Yellow Sea, China. Int. Biodet. Biodeg. 107, 62–69. 10.1016/j.ibiod.2015.10.022

